# Training‐Free Regulation of Grasping by Intracortical Tactile Feedback Designed via S1‐M1 Communication

**DOI:** 10.1002/advs.202503011

**Published:** 2025-06-30

**Authors:** Qi Zhang, Bing Liu, Zhemeng Wang, Jiayue Zhou, Xingyi Yang, Qian Zhou, Yuwei Zhao, Siwei Li, Jin Zhou, Changyong Wang

**Affiliations:** ^1^ Beijing Institute of Basic Medical Sciences Beijing 100850 China; ^2^ School of Life Science and Technology Harbin Institute of Technology Harbin 150001 China; ^3^ Institute of Automation Chinese Academy of Sciences Beijing 100190 China; ^4^ Peking University Beijing 100871 China; ^5^ Capital Medical University Beijing 100038 China; ^6^ Beijing Simulation Center Beijing 100039 China

**Keywords:** brain‐machine interface, grasping, intracortical microstimulation, sensorimotor cortex, tactile feedback

## Abstract

Tactile feedback is essential for grip force control when operating a neuroprosthesis. Due to limited knowledge of cortical sensorimotor coordination, artificial feedback is mostly counterintuitive, requiring training to be associated with grasping behaviors. The current study investigates sensorimotor communication by recording neural activities from the primary sensory cortex (S1) and the primary motor cortex (M1) while macaques grasp targets of various textures and loads. Intracortical micro‐stimulation is also delivered to S1 to validate the intervention of sensorimotor communication in grasping. The findings identify an S1→M1 functional pathway through which tactile information is transferred. The pathway is shared by both natural and artificial neural propagations. Moreover, it is demonstrated that sensory and motor decoding of neural activities in M1, as well as the actual grip force, are modulated by stimulation designed via S1→M1 communication, without prior training. The work provides a biomimetic strategy to design intuitive haptic feedback for brain‐machine interfaces utilizing the S1→M1 pathway.

## Graphical abstract

During object grasping in macaques, information flow between the primary sensory cortex (S1) and the primary motor cortex (M1) is modulated by tactile properties of the target. Intracortical micro‐stimulation in S1 reliably evokes M1 responses that resemble those naturally generated during grasping under specific tactile conditions. Biomimetic stimulation patterns designed to reproduce these M1 responses can effectively modulate both neural and behavioral grasping responses without prior training.

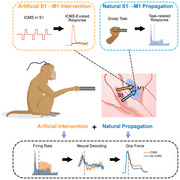

## 1 Introduction

Grasp force control is a fundamental function through which primates interact with objects in the external environment. In applications in which neuroprosthetic hands were operated by brain‐machine interfaces (BMIs), the precise control of grip force depends on extrinsic sensory feedback regarding the target's tactile properties such as roughness and weight. Recent studies of bidirectional BMIs with sensory input loops have demonstrated that, neuroprosthesis with tactile feedback delivered by intracortical microstimulation (ICMS) not only enables the tactile perception of objects,^[^ ^1^, ^4^ ^]^ but also improves the efficiency of grasping tasks.^[^ ^5^, ^6^ ^]^ While ICMS in the primary sensory cortex (S1) is capable of inducing artificial sensations,^[^ ^7^, ^11^ ^]^ current sensory feedback for bidirectional BMIs is non‐natural, requiring prior training to link the stimulation with grasping behaviors, resulting in more time‐consuming and effort‐consuming regulations compared to authentic sensation.^[^ ^12^ ^]^ Designing biomimetic sensations to achieve spontaneous grasping modulation is challenging, as the pathways through which sensory perception reorganizes motion remain unclear.

Previous studies have investigated cortical pathways for hand sensation and motion separately in non‐human primates. Tactile sensation is known to be projected from the thalamus to the primary sensory cortex,^[^ ^13^, ^14^ ^]^ where it is richly encoded,^[^ ^15^, ^19^ ^]^ and subsequently propagates to areas such as the secondary sensory cortex (S2) and the dorsal posterior parietal cortex, where it contributes to advanced cognitive functions such as decision‐making,^[^ ^20^, ^21^ ^]^ Recently, tactile sensations have also been found to be encoded in additional nuclei, such as the amygdala and the cuneate nucleus.^[^ ^22^, ^23^ ^]^ For motor control in grasping, parameters of motion are encoded in the circuit of cortical regions including the primary motor cortex (M1), the premotor cortex, and the posterior parietal cortex,^[^ ^24^, ^29^ ^]^

Although sensory and motor circuits were largely investigated independently, recent research revealed functional overlaps between S1 and M1,^[^ ^30^, ^34^ ^]^ implying communications between the two areas.^[^ ^35^, ^40^ ^]^ Research in rodents proposed that S1 conveys proprioceptive information to M1 in addition to the classical tactile projection from S1 to S2.^[^ ^36^ ^]^ For studies on human and non‐human primates, the functional pathway between S1 and M1 was identified by modulating M1 responses with either invasive^[^ ^41^, ^43^ ^]^ or non‐invasive^[^ ^44^, ^45^ ^]^ stimulation in S1. However, the flow of tactile properties interacting between sensory and motor cortices during grasping remains unknown.

Moreover, in bidirectional BMIs for non‐human primates, sensory feedback was mostly delivered to S1 electrodes representing tactile sensation, overlooking the pathway of tactile information propagation. Given that thalamocortical connections could be used to mimic authentic S1 responses with stimulation in the thalamus,^[^ ^46^, ^48^ ^]^ it is inferred that within a neural pathway of sensations, encoding stimulation of upstream regions aiming at eliciting natural tactile responses in downstream regions, may facilitate the induction of authentic tactile perceptions. Therefore, if a direct pathway exists for conveying tactile information between S1 and M1, it would be possible to simulate the sensorimotor interaction through biomimetic stimulation in S1, enabling rapid and unconscious regulation of sensorimotor coordination with no prior training required.

We hypothesized that: (1) during grasping, a functional pathway exists between S1 and M1, propagating tactile sensations (e.g., surface texture and load of the target) from sensory to motor circuits to modulate grasp behaviors (e.g., grip force); (2) by intervening in S1→M1 communication with ICMS, both neural and behavioral characteristics of grasping could be modulated without training the animal to recognize the stimulus. We tested our hypothesis by experiments in which two macaques grasped objects under various tactile conditions. First, we quantified tactile information transferred between S1 and M1 during grasping through functional connectivity analysis. Secondly, we verified the capability of S1 ICMS in directionally modulating tactile representations in M1. Finally, we validated the role of S1→M1 communication in regulating grip force decoding as well as actual grasping behaviors.

## 2 Results

**Figure 1:**
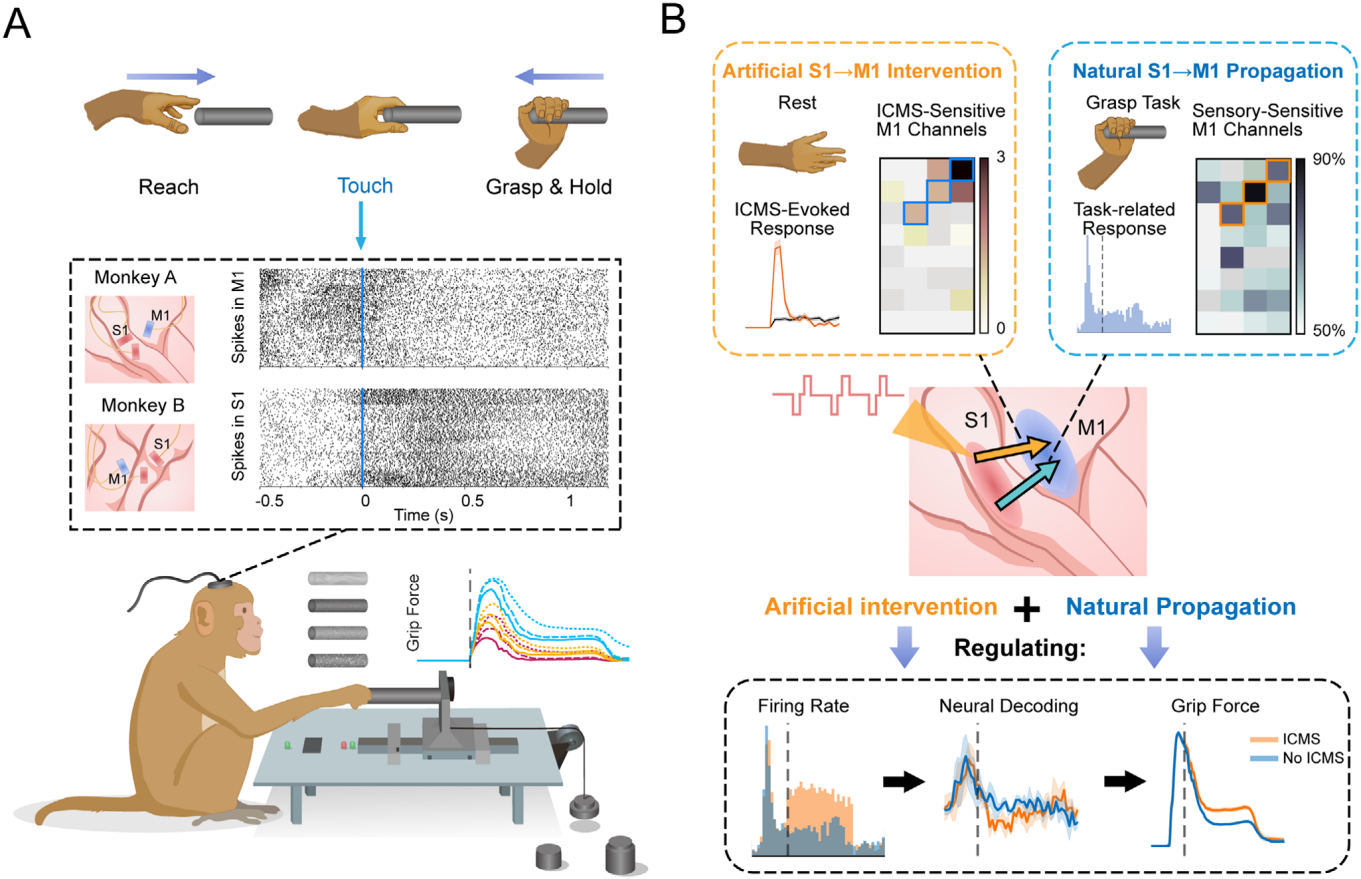
Schematics of the main contents. A) Schematic of the experimental paradigm and implantations. The monkeys were required to grasp a cylindrical force dynamometer and maintain holding for 700 ms to receive a reward. The experiment included four types of textures (P36 sandpaper, P400 sandpaper, P7000 sandpaper, waterproof fabric) and three levels of loads (400, 550, and 700 g). Each monkey had one array of electrodes implanted in M1 and two implanted in S1. B) Schematic of the main contents. The current research identified a shared functional pathway from S1 to M1 for both natural propagation and artificial intervention in tactile sensations. By ICMS encoded via this cortical pathway, training‐free modulations on M1 responses, performances of neural decoding, and actual grasp behaviors were achieved.

The current study consists of results from three sections of experiments. The first section aimed to illustrate the neural coding of different textures and loads in S1 and M1 during grasping, and to identify the functional pathway conveying tactile properties. In this section, animals were required to complete a target grasping task under various conditions of textures and loads, while neural responses of S1 and M1 were simultaneously recorded. The second section aimed to further test the functional connectivity between M1 and S1 by exploring the responsiveness of M1 to rest‐state ICMS in S1. Thirdly, we evaluated the effects of S1 stimulation on both neural response in M1 and force modulation during grasping. The animals were required to complete grasping tasks accompanied by ICMS in S1. Two macaques participated in the current study and completed data collection for all experiments (**Figure** **1**A,B; Figure S1A, Supporting Information).

### 2.1 Temporal Effect of Texture and Load in Sensorimotor Cortex

First, we explored how textures and loads were encoded in the sensorimotor cortex. A total of 16 sessions of datasets were collected in the first experimental section (8 from monkey A and 8 from monkey B), including data from 1865 units, 835 from M1, and 1030 from S1. For each unit, two‐way ANOVA was conducted on the moving average of firing rate to compute the continuous effect of textures and loads, quantified by η^2^. Due to the heterogeneity of neurons in the somatosensory cortex, some units did not participate in representing tactile conditions. Therefore, units that were not sensitive to textures nor loads were dropped according to the corrected p‐value (see more details in Experimental Section). 610 units from M1 and 655 from S1 were included in the analysis. The temporal results were aligned to the moment of touching the target (0 ms).

**Figure 2:**
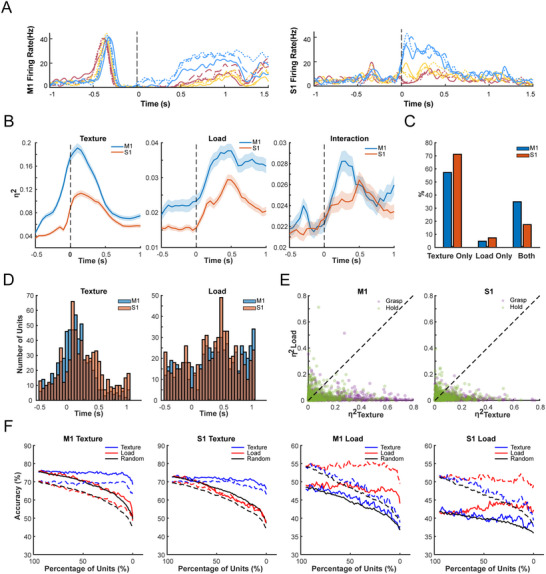
Temporal Tuning and Functional Compartment of Textures and Loads in M1 and S1 during Grasping. A) Firing rates of sample units in M1 and S1 during grasping, aligned to the moment of touching the target. Textures are distinguished by color (blue for P7000 sandpaper, yellow for P400 sandpaper, pink for P36 sandpaper), while loads are distinguished by line styles (solid line for 400 g, dashed line for 550 g, dash‐dot line for 700 g). B) Main effects of textures (left), loads (middle), and their interaction (right) as functions of time (*n* = 610 units for M1 and *n* = 655 units for S1). The shaded areas represent the standard deviation obtained through bootstrap sampling (same for all plots with shadows in this study). C) Proportion of units affected by textures and loads in M1 and S1 during ‐50–500 ms after touching (covering the stages of T and GH). M1 (34.92%) achieves a greater proportion of units to respond to both texture and load conditions than S1(17.44%), whereas units in S1 tend to react to either textures or loads. D) Distribution of peak latencies for effects of textures and loads. S1 shows concentrated peak latencies for textures (the left panel, ‐50–150 ms) and loads (the right panel, 350–550 ms), while latencies in M1 are relatively dispersed. E) Tuning of neuronal activity by textures and loads in stages of T and GH. Units were predominantly tuned by textures during stage T while tuned by loads during stage GH. F) Population classification accuracy for textures and loads as functions of the number of units. The solid lines represent decoding accuracy during touching, while the dashed lines represent accuracy during grasping and holding.

A temporal delay between effects of textures and loads was observed in both M1 and S1 (**Figure** **2**B). Event‐locked effects were shown among S1 units, with texture effects most frequently peaking at ‐50–150 ms, when the monkey initially touched the target. Effects of load most frequently peaked at 350–550 ms, at which the monkey was in the transition between grasping and holding (Figure 2D). In M1, although tactile effects were not locked at a specific time point, the peak latencies for texture effects were earlier than load. This temporal delay was also confirmed in population response by building a Support Vector Machine (SVM) decoder to classify tactile conditions on population of units. Similarly, the peak latency for the performance of texture classification (M1: ‐50–150 ms; S1: 0–200 ms) was found to be earlier than that of load (M1/S1: 350–550 ms, Figure S1C, Supporting Information). We defined stages Touching (T, ‐50–150 ms) and Grasping&Holding (GH, 350–550 ms) according to the behavior of monkeys. Units in both M1 and S1 were influenced more intensely by textures during stage T compared to stage GH (M1: *p* < 0.001; S1: *p* < 0.01, Wilcoxon Signed‐Rank test), but were affected more intensely by loads during stage GH, conversely (M1: *p* < 0.001; S1: *p* < 0.001, Figure 2E). These results suggested that sensorimotor cortex was affected mostly by textures during surface touching, but was strongly affected by loads in later stages of grasping and holding when monkeys maintained grasping according to load levels.

### 2.2 Compartment of Texture and Load Representation in M1 and S1

Functions of encoding tactile conditions were compared between units in M1 and S1. A greater overall proportion of units responded to both textures and loads in M1 (34.92%) than that in S1 (17.44%, Figure 2C; Figure S1B, Supporting Information). In addition, a peak of texture × load interaction (200–400 ms) was observed for units in M1 between the peak effects of textures and loads, but was not observed in S1 (Figure 2B), suggesting a gradual transition from representing textures to loads for M1 units, whereas units in S1 represented a relatively demixed pattern of textures and loads. To verify whether the functions of textures and loads were independent in terms of population response, we performed classification with varying numbers of units. Units were dropped out in the order of texture or load sensitivity, and also in random order for comparison. A small group of 8% texture‐sensitive units in M1 (S1: 10%), or 8% of load‐sensitive units in M1 (S1: 8%) were required to achieve 95% of the top accuracy if units were dropped in the corresponding order to textures and loads (Figure 2F). Whereas when dropping in the non‐corresponding order, the accuracy curve was similar to when dropping in the random order. The above results suggested that comparable classification performance to the intact population could be achieved by a small subset of units, where textures and loads were represented independently.

### 2.3 The Information Stream from S1 to M1 is Tactile‐related during Grasping

**Figure 3:**
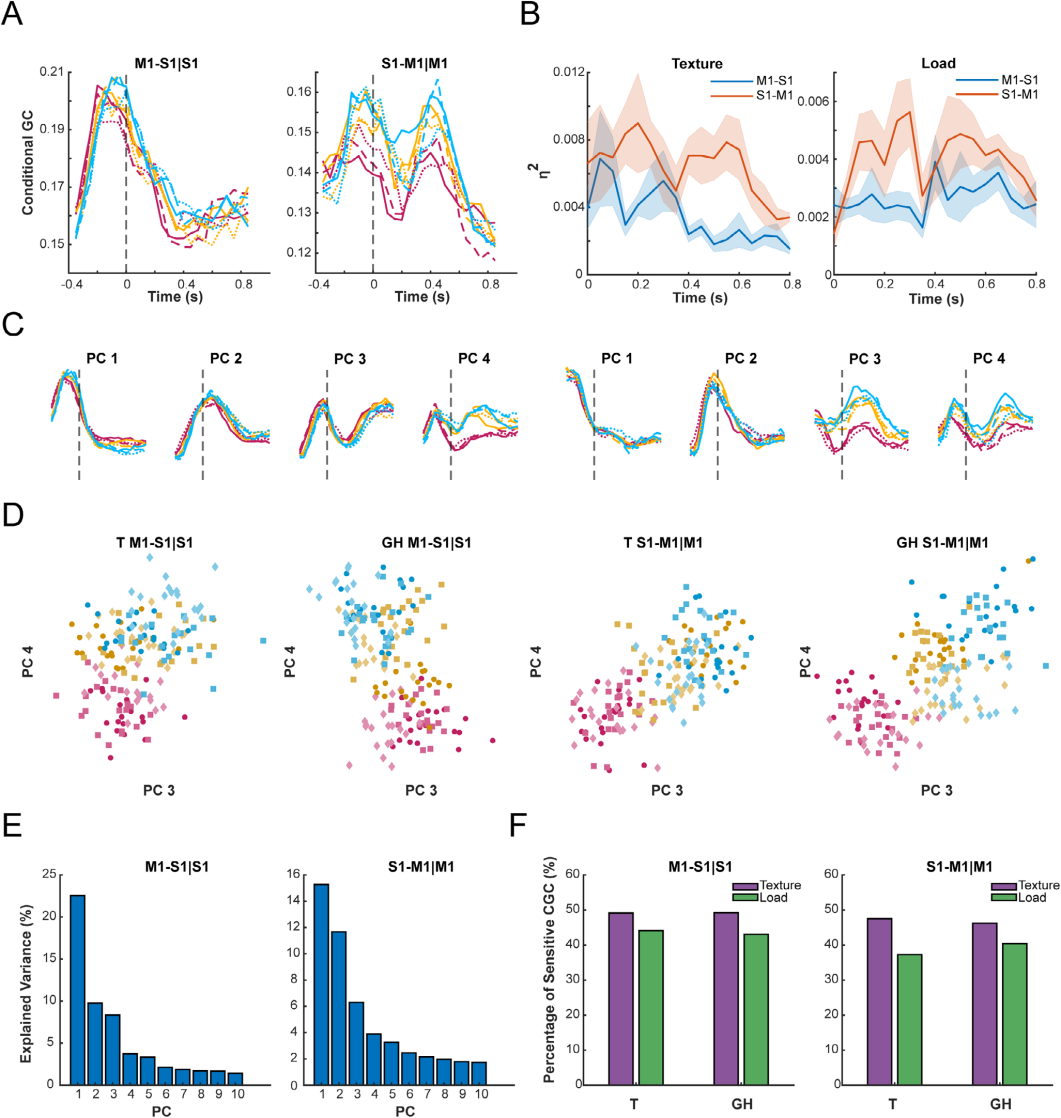
Information stream from S1 to M1 is tactile‐related during grasping. A) The mean conditional Granger causalities (CGCs) for all 16 sessions as functions of time. The colors represent textures of varying roughness, with blue indicating the smoothest texture and pink the indicating roughest. Line styles represent load levels: solid line for 700 g, dashed line for 550 g, and dash‐dot line for 400 g. B) Continuous effects of textures (left) and loads (right) on CGC (*n* = 610 pairs for M1→S1|S1, *n* = 659 pairs for S1→M1|M1). Effects of tactile conditions on GCG show bimodal pattern peaking at 50–350 and 350–650 ms. The shaded areas represent the standard deviation obtained through bootstrap sampling. C) The first four principal components (PC) after PCA. The S1→M1|M1 CGC shows sensitivity to texture levels from PC3, and sensitivity to load levels in PC4. For the M1→S1|S1 CGC, effect of textures begins to manifest from PC4 onwards. D) Projection of CGC on PC3 and PC4 during stages T and GH. Colors represent textures of varying roughness (same with A)), while the shapes of scatters represent levels of load: dots for 400 g, squares for 550 g, and diamonds for 700 g. Each observation reflects CGC from resampled trials (20% trials resampled for 20 times). E) Variance explained by each PC. 44.36% of the variance for CGC of M1→S1|S1 is collectively explained by the first four PCs, while 37.13% is explained for CGC of S1→M1|M1. F) Proportions of unit pairs sensitive to texture and load conditions in stages T and GH. CGC M1→S1|S1: Texture: 49.13% for stage T, 49.21% for GH; Load: 44.09% for T, 43.06% for GH; CGC S1→M1|M1: Texture: 47.51% for T, 46.23% for GH; Load: 37.27% for T, 40.39% for GH.

Non‐parametric conditional Granger causality (CGC) was employed to investigate the bidirectional information transfer between S1 and M1.^[^ ^49^ ^]^ The influence of other M1 units was considered when computing CGC from S1 to M1 (S1→M1|M1), and vice versa (M1→S1|S1). Continuous CGCs were applied with 300 ms sliding time windows on 1600 pairs of S1 and M1 units from 16 datasets. The significance of CGC was examined with a spike‐jittered method^[^ ^50^ ^]^ for both stages T (‐100–200 ms, matching the 300 ms window length for CGC) and GH (300–600 ms). Unit pairs that were not significant in neither stage were excluded, resulting in a total of 923 pairs of M1→S1|S1 CGCs and 659 pairs of S1→M1|M1 CGCs. The average M1→S1|S1 CGCs peaked before reaching the target and subsequently decreased during the grasping process. In contrast, S1→M1|M1 CGCs presented a second peak between at 300–600 ms, suggesting that information was transferred from S1 to M1 over the late process of grasping and holding (**Figure** **3**A).

Next, we tested whether the information transferred from S1 to M1 carried information of textures and loads. For each dataset, two‐way ANOVAs were adopted to test the effect of textures and loads on unit pairs with significant CGC (Figure 3B). As a result, the main effect of textures and loads on S1→M1|M1 CGCs exhibited a bimodal pattern, indicating that during the early grasping and later holding processes, the propagation from S1 to M1 conveyed tactile information, which might regulate grasping behaviors. Given the heterogeneity of unit pairs, we tested the tactile effect on each pair of units. During stage T, 49.13% of M1→S1|S1 CGCs were significantly influenced by textures, while 44.09% were influenced by loads (Figure 3F). For S1→M1|M1 CGC, 47.51% were sensitive to textures and 37.27% to loads. A similar pattern was obtained during stage GH (M1→S1 texture: 49.21%; load: 43.06%; S1→M1 texture: 46.23%; load: 40.39%). Finally, we verified tactile effects via Principal Component Analysis (PCA, Figure 3C–E). For S1→M1|M1 CGCs, effects of textures were observed on principal components (PCs) 3 and 4, while load effects appeared on PC4 during late phase of holding (Figure 3C). Projections of resampled CGCs on PC3 and PC4 revealed distinguishable conditions of textures and loads in stage GH (Figure 3D). Similar results were found for CGCs with all 1600 unit pairs (Figure S2, Supporting Information). In conclusion, our results not only revealed significant neural stream from S1 to M1 but also demonstrated that the information transferred was tactile‐related.

### 2.4 Stimulations in S1 Evoke Pulse‐related Responses in M1

**Figure 4:**
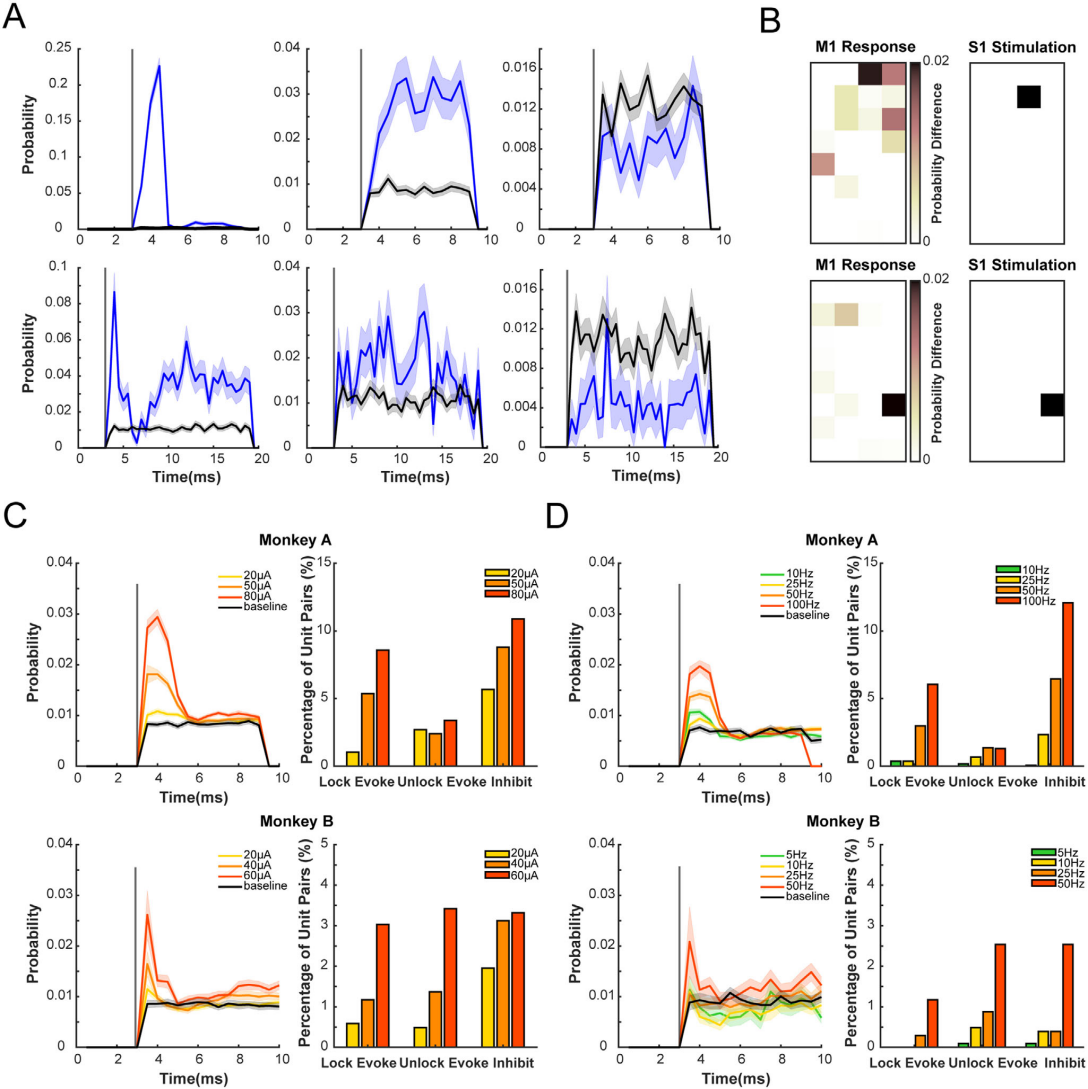
ICMS in S1 evokes pulse‐related response in M1. A) Examples of the responses of M1 channels to ICMS in S1. Three types of M1 responses were identified: phase‐locked elicitation (left), non‐phase‐locked elicitations (middle), and inhibitions (right). Responses were quantified as firing probability. ICMS‐evoked responses are present in blue, and baseline responses are in black. The top row displays data from monkey A, and the bottom row displays data from monkey B. The shaded areas represent the standard deviation obtained through bootstrap sampling. B) An example of M1 responses to ICMS at different sites in S1 from monkey A. The spatial pattern of M1 response (left) was adjusted by varying locations of stimulated S1 sites (right). M1 responses are quantified as the difference between the post‐stimulation firing probability and the baseline firing probability without stimulation. C) Effects of ICMS amplitude on M1 responses. The right panel shows the proportion of significant stimulation‐response pairs, and the left panel shows the magnitude of phase‐locked elicitations. Data from monkey A is shown in the top row, and data from monkey B are shown in the bottom row. The shaded areas represent the standard deviation obtained through bootstrap sampling. D) Similar to C) but presents effects of ICMS frequency on M1 responses.

Given the information propagation from S1 to M1, we assumed a direct functional pathway through which ICMS in S1 could induce pulse‐related responses in M1. Instead of units after manual sorting, we took unsorted multi‐unit data from each M1 channel into analysis. Data from 256 M1 channels (32 channels * 8 sessions) were recorded from 8 rest‐state sessions (4 for monkey A and 4 for monkey B). M1 responses were categorized into three types: phase‐locked elicitation, non‐phase‐locked elicitation, and inhibition (**Figure** **4**A). Specifically, phase‐locked elicitations implied direct links between M1 and S1.^[^ ^43^ ^]^ For monkey B, 19% of M1 channels exhibited phase‐locked activation in response to stimulation of at least one S1 site, 29% channels were activated in non‐phase‐locked patterns, while 30% were inhibited. Although M1 activities for monkey A were relatively inhibitory, a proportion of channels were still activated (phase‐locked elicitation: 28%; non‐phase‐locked elicitation: 24%; inhibition: 66%). Furthermore, the variation in stimulation sites in S1 resulted in unique spatial patterns of M1 responses (Figure 4B; Figure S3C, Supporting Information), emphasizing the importance of the stimulated location even within the small area of the implanted array.

Moreover, effects of ICMS amplitude and frequency on M1 responses were examined. Three levels of amplitude and four levels of frequency were set for each animal (monkey A: 20/50/80 µA, 10/25/50/100 Hz; monkey B: 20/40/60 µA, 5/10/25/50 Hz). For both monkeys, the number of all three types of responding pairs increased with the level of amplitude (Figure 4C). Additionally, the magnitude of the elicited M1 response—especially phase‐locked elicitations—increased with the level of amplitude (Figure 4C; Figure S3A, Supporting Information). In terms of frequency, although not varied under 25 Hz, the number and magnitude of M1 responses over 50 Hz were markedly greater than those at lower frequencies (Figure 4D; Figure S3B, Supporting Information). Overall, within the experimental settings, both frequency and amplitude positively influenced the elicitation in M1.

### 2.5 Pulse‐Evoked M1 Responses Depend on Tactile Sensations

The effect of ICMS on M1 responses during grasping was investigated. Experimental conditions in each dataset were crossed by two levels of textures or loads and two different stimulation sites in S1. We chose stimulation locations as the variable for ICMS because spatially differed stimulations were likely to achieve varied patterns of modulation on M1 responses. To decide stimulation sites for the experiment, we first selected M1 channels sensitive to tactile levels and then identified S1 sites that could modulate the selected M1 channels (see Experimental Section).

**Figure 5:**
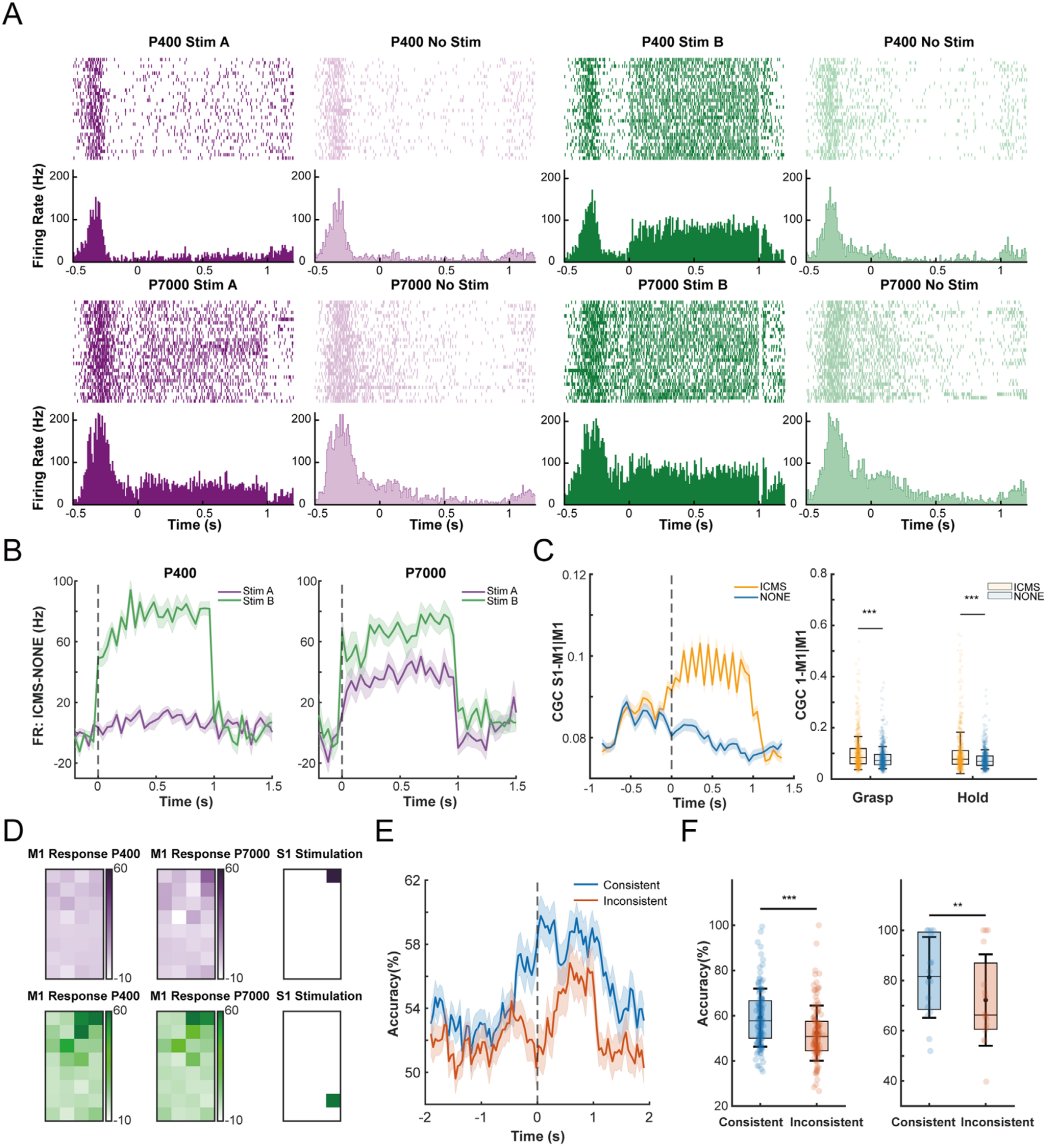
Pulse‐evoked M1 responses depend on tactile sensations. A) Raster plot of a sample M1 channel under different stimulation conditions (stimulus site A, B, and no stimulus) and tactile conditions (sandpapers P400 and P7000). This channel exhibits both texture and stimulus effects within the 0–1 s period following stimulus onset, with a significant interactions between these factors. B) Two examples of M1 channels with significant interaction between tactile levels and ICMS sites. The left two panels are from one channel, while the right two are from another. The responses under the two different stimulations varied between conditions of P400 and P7000 sandpapers. C) Effect of ICMS in S1 on CGC S1→M1|M1. The left panel shows CGC S1→M1|M1 over time under ICMS and no‐stimulation conditions across 781 S1–M1 channel pairs. The shaded areas represent the standard deviation obtained through bootstrap sampling. The right panel presents statistical comparisons between ICMS and no‐stimulation conditions during the grasping (0–280 ms) and holding (400–680 ms) phases, both showing significant increases under ICMS (*p* < 0.001, Wilcoxon signed‐rank test, *n* = 781 channel pairs). D) Spatial pattern of ICMS‐evoked M1 response under different tactile levels. The left panels display M1 responses for P400 texture condition, while the middle panels display responses for P7000 texture condition. The right panels illustrate stimulation locations in S1. E) Performance of classifying stimulation sites by channels sensitive to the interaction between tactile and stimulation conditions (*n* = 61 channels). Each observation corresponds to one channel in M1. The shaded areas represent the standard deviation obtained through bootstrap sampling. F) Boxplots of average performance in classifying stimulation sites within 0–400 ms after stimulus onset. The accuracies of consistent training and testing sets are greater than those of inconsistent sets for both single‐channel classifiers (left, *p* < 0.001, Wilcoxon Signed‐rank test, *n* = 61 channels) and population classifiers (right, *p* < 0.01, *n* = 16 blocks). n.s.: *p* > 0.05; *p < 0.05; **p < 0.01; ***p < 0.001.

We first tested whether ICMS delivered to S1 could modulate the causal relationship from S1 to M1. Due to hardware limitations that precluded simultaneous recording from stimulated S1 channels, the broader effects on S1→M1 information propagation were evaluated by analyzing data recorded from the neighboring S1 array. For each dataset, channels with top 10 firing rates in S1 and M1 were selected for analysis (with one dataset including only 9 channels due to limited firing rates). In total, 781 S1‐M1 channel pairs across 8 datasets were included for conditional Granger causality analysis. It was observed that ICMS increased CGC S1→M1|M1 during both the grasping (0–300 ms) and holding (400–700 ms, *p* < 0.001), indicating that stimulation in S1 can enhance the global information flow from S1 to M1 (**Figure** **5**C). Notably, the two stimulation sites did not differ significantly in their modulation of CGC S1→M1|M1 (*p* > 0.05, Figure S4A‐C, Supporting Information), possibly due to the use of S1 data from neighboring electrodes rather than the directly stimulated ones, which may have limited the sensitivity to stimulation‐specific neural projections.

Although ICMS‐induced responses were known to depend on motor tasks,^[^ ^43^ ^]^ the interaction between stimulation and tactile levels has not been verified. Hence, we identified effects of stimulation and tactile conditions on M1 channels during grasping. As a result, 23.83% M1 channels (61 of 256 channels, 38 for sessions of textures, and 23 for sessions of loads) exhibited significant interactions of stimulation sites and tactile levels on firing rates within 0–400 ms after stimulation onset (*p* < 0.05, Figure 5A,B). If an interaction exists, pulse‐evoked responses should differ across tactile levels. Therefore, an SVM decoder was used to classify the two ICMS sites on channels with significant interactions. The decoder was trained on data from one tactile level and tested on data from both tactile levels. Results indicated that the accuracy was significantly higher when the training and testing sets were from the same tactile level compared with inconsistent levels (*p* < 0.001, Figure 5D‐E; Figure S5A,B, Supporting Information), demonstrating that the impact of stimulation on neural responses is modulated by tactile levels. Consistent results were found for population classification (*p* < 0.01, Figure 5D; Figure 4C), confirming the existence of sensory‐stimulation interaction from a population perspective. Therefore, our findings indicated a shared S1→M1 functional pathway for both natural tactile propagation and artificial intervention by ICMS. It is possible that the transfer of tactile information could be modulated by a specifically designed stimulus in S1.

### 2.6 Tactile Sensory Encoding in M1 is Directionally Modulated through the S1→M1 Pathway

**Figure 6:**
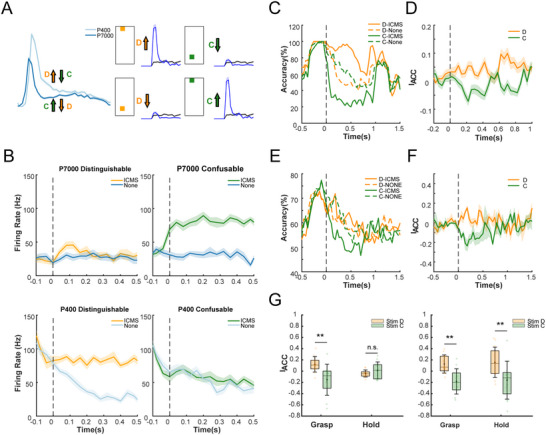
Tactile sensory encoding in M1 is directionally modulated through the S1→M1 pathway. A) Schematic of M1 responses modulated by spatially encoded S1 stimulation. Responses became closer to each other under stimulus C but further under stimulus D. B) A sample channel that was modulated directionally by stimuli C and D. The normalized firing rates for P7000 were increased by stimulus C, resulting in similar responses to that for P400. Whereas the normalized firing rates for P400 were increased by stimulus D, resulting in distinct responses compared to that for P7000. The shaded areas represent the standard deviation obtained through bootstrap sampling. C) Performance of the sample channel in classifying tactile levels. D) Index of difference (I_ACC_) for all tactile‐sensitive channels in M1 (*n* = 82). The shaded areas represent the standard deviation obtained through bootstrap sampling. E) Performance of neural population in classifying tactile levels. F) Similar to D) but presents the I_ACC_ for population classification of all sessions (*n* = 8). G) IACC for sessions of textures (left) and loads (right) during grasping (0–280 ms) and holding (400–680 ms). Stimulus D surpassed C in improving load classification during both grasping (*p* < 0.01) and holding (*p* < 0.01), but only during grasping (*p* < 0.01) for texture classification (Wilcoxon Signed‐rank test, *n* = 12 time points, n.s.: *p* > 0.05; *p < 0.05; **p < 0.01; ***p < 0.001).

We then tested the feasibility of modulating tactile representations in M1 by ICMS delivered to specially selected sites in S1 corresponding to the two tactile levels. Two types of stimuli pairs were designed: Distinguishable pairs (D), which aided in the distinction of neural activity between the two tactile levels; and Confusable pairs (C), which tended to blur the difference in tactile levels (see Experimental Section). For example, in the M1 channels illustrated in **Figure** **6**A, the firing rate for P400 sandpaper was greater than that for P7000 sandpaper. Thus, when grasping targets of P400 sandpaper, stimulus C would suppress the neural response of that channel, leading to a lower firing rate alike that when grasping P7000 sandpaper. Conversely, stimulus D would activate the neural response, enhancing the gap between the two tactile levels. Data from 8 sessions were analyzed (4 from monkey A and 4 from monkey B).

An SVM classifier trained with single‐channel responses was applied to classify the tactile levels of trials under stimuli C and D (Figure 6C). We used the index of accuracy difference between ICMS and non‐ICMS trials to describe the effect of ICMS in modulating the tactile representation (I_ACC_, see Experimental Section). Only channels with significant effects of textures or loads were included in the analysis, as it is meaningless to include channels unresponsive to tactile conditions in tactile classification. The positive I_ACC_ for stimulus D (0.034) during 0–400 ms after stimulation onset indicated an increase in the gap between tactile levels, whereas the negative I_ACC_ for stimulus C (−0.027) indicated a reduction in the gap (Figure 6D). To further validate the directional modulation on population response, we expanded the input of the classifier to all M1 channels within a session. The average classification accuracy for the trials with stimulus D was slightly greater compared to the no‐stimulation control group, while trials with stimulus C exhibited a decline in average classification accuracy (Figure 6E‐F). Additionally, effects of proposed stimuli on textures and loads were examined respectively during grasping (0–,280 ms) and holding (400–680 ms). For sessions of loads, the I_ACC_ for stimulus D was significantly greater than that for stimulus C during both grasping and holding (Grasping: p < 0.01; Holding: *p* < 0.01, Figure 6G; Figure S6A,B, Supporting Information). However, for sessions of textures, the I_ACC_ for stimulus D was significantly greater than that for stimulus C only during grasping (*p* < 0.01) but not holding (*p* > 0.05). Given that textures achieved the greatest effect at the stage of touching before grasping, the stimulation during holding might not effectively intervene in neural representation. Finally, to assess the attenuation of ICMS effects due to neural adaptation, we quantified the average classification accuracy across the 30 trials within each block. Results showed that stimulation at sites C and D consistently influenced decoding accuracy throughout all 30 trials, indicating that the effects of stimulation within each block were not diminished by short‐term neural adaptation (Figure S7B, Supporting Information). Overall, our results indicated that stimuli on selected sites in S1 achieved directional modulation of tactile encoding for both single‐channel and population activities in M1.

### 2.7 Sensory Feedback Encoded via S1→M1 Interaction Directionally Regulates Motor Decoding

ICMS in S1 leads to mutations in M1 response, thereby regulates the performance of motor decoding. In applications involving brain‐controlled robotic hand grasping, it is desirable to design haptic feedback in S1 that adjusts the decoded grip force to match the target's tactile property, with minimal effort required from the operator. Given that grip force is affected by texture and load conditions, it was hypothesized that stimuli designed to distinguish tactile levels (stimulus D) would amplify the difference in force decoding between two tactile conditions, whereas stimuli designed to confuse tactile levels (stimulus C) might reduce or reverse the difference.

**Figure 7:**
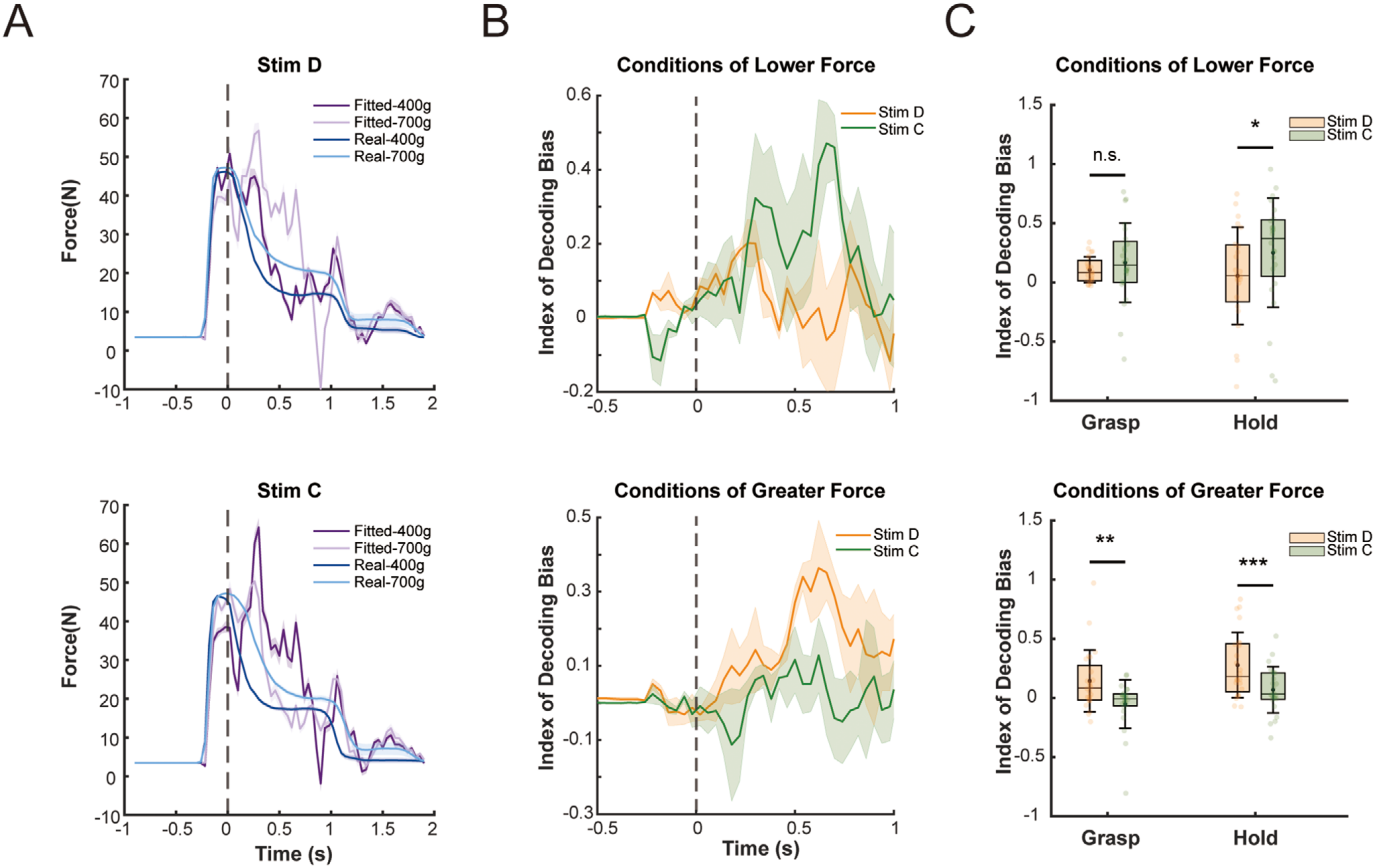
Sensory feedbacks encoded via S1→M1 interaction directionally regulate motor decoding. A) Sample blocks of grip force decoding under stimulus D (upper panel) and stimulus C (lower panel). Stimulus D amplifies decoded grip force under the greater‐force condition (700 g), enhancing the separability between tactile conditions. In contrast, stimulus C increases decoded force under the lower‐force condition (400 g), disrupting the tactile separability. The shaded areas represent the standard deviation obtained through bootstrap sampling. B) Bias for grip force decoding across all sessions (*n* = 8). The upper panel shows force bias for conditions of lower grip force, while the lower panel shows force bias for conditions of greater force. For conditions of lower grip force (upper), stimulus C leads to positive biases, while stimulus D leads to negative biases during holding. Conversed pattern is shown for conditions of greater force (lower). The shaded areas represent the standard deviation obtained through bootstrap sampling. C) Bias for grip force decoding during Grasping (0–280 ms) and Holding (400–680 ms). The upper panel shows force bias for conditions of lower grip force, while the lower panel shows force bias for conditions of greater force (Wilcoxon Signed‐rank test, *n* = 16 blocks, n.s.: *p* > 0.05; *p < 0.05; **p < 0.01; ***p < 0.001).

We applied a Support Vector Regression (SVR) model to fit grip force with neural activity in M1 under stimuli C and D. The model was trained with trials without ICMS. It was observed that stimulus D induced biases aligned with the actual trends of grip force across tactile conditions, while stimulus C caused opposing biases (**Figure** **7**A). We proposed the index of decoding bias to quantify the effect of designed stimuli on decoding modulation (see Methods). For conditions requiring lower grip force, such as coarse textures or low loads, stimulus C induced a positive bias in force decoding during holding compared to stimulus D, which caused a negative bias. Conversely, for conditions requiring greater grip force, stimulus D induced a significant positive bias during grasping and holding compared to stimulus C, which caused a negative bias (Figure 7B,C). To assess whether neural adaptation attenuated the effects of ICMS, we quantified the average grip force decoding bias across the 30 trials within each block. Stimulation at sites C and D consistently induced a stable shift in grip force decoding throughout the 30 trials, indicating that the stimulation effects within each block were not diminished by short‐term neural adaptation (Figure S7A, Supporting Information). Our findings demonstrated that tactile feedback encoded via S1→M1 communication could directionally regulate grip force decoding, offering potential for effortless force control in operating robotic hand grasping.

### 2.8 Actual Grip Force is Modulated through S1→M1 Interaction without Training

Visually, grip force appeared to be affected by ICMS at 300 ms after the onset of stimulation (Figure S8, Supporting Information). Hence, we compared the average force during 300–,700 ms (after 700 ms holding was no longer required for reward). Among the 32 blocks with either stimulation C or D, 18 (56.25%) revealed an effect of ICMS on grip force.

**Figure 8:**
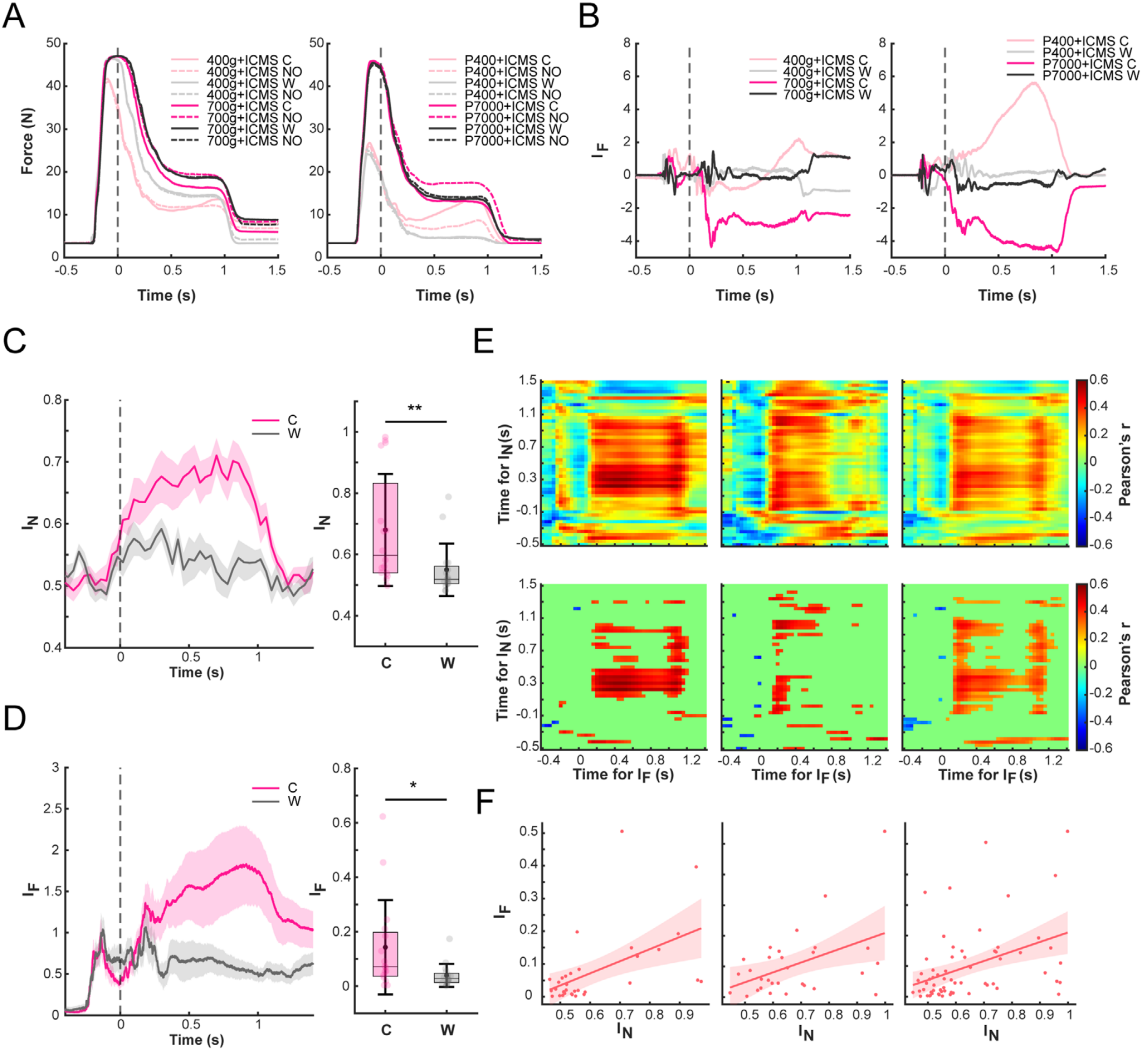
Actual grip force is modulated through S1→M1 interaction without training. A) Trial‐averaged grip forces for two sample sessions of load (left) and texture (right) levels from monkey A. Effects of ICMS on force appear from 300 to 1000 ms after the onset of stimulation. See Figure S9 (Supporting Information) for grip forces of the remaining sessions. B) Trial‐averaged index of difference for grip force (I_F_) for two sample sessions of load (left) and texture (right) levels. C) Neural modulation by stimuli W and C, reflected by the classification accuracy for ICMS/no ICMS (I_N_). The left panel shows continuous I_N_, while the right panel shows averaged I_N_ from 300 to 700 ms post‐stimulation for each block, indicating stimulus C led to greater neural modulation than W (*p* < 0.01, *n* = 16, Wilcoxon Signed‐rank test, n.s.: *p* > 0.05; *p < 0.05; **p < 0.01; ***p < 0.001). The shaded areas represent the standard deviation obtained through bootstrap sampling. D) Behavioral modulation by stimuli W and C, reflected by the index of difference on grip force (I_F_). The left panel shows continuous I_F_, while the right panel shows averaged IF from 300 to 700 ms post‐stimulation for each block, indicating stimulus C led to greater behavioral modulation than W (*p* < 0.01, *n* = 16, Wilcoxon Signed‐rank test, n.s.: *p* > 0.05; *p < 0.05; **p < 0.01; ***p < 0.001). The shaded areas represent the standard deviation obtained through bootstrap sampling. E) Correlation matrices between I_N_ and I_F_. Matrices from left to right regard correlations of 32 observations (blocks) from 8 sessions of stimuli C and W, correlations of 32 observations (blocks) from 8 sessions of stimuli C and D, and correlation of 64 observations from the total 16 sessions of C/D and C/W, respectively. The bottom row shows same results but only significant correlations (*p* < 0.05) are shown. F) Correlation between the mean IF during 200–700 ms and the mean I_N_ during 140–500 ms (panels as described in (E) left: *r* = 0.5123, *p* < 0.01, *n* = 32 blocks; middle: *r* = 0.4541, *p* < 0.05, *n* = 30 blocks; right: *r* = 0.4092, *p* < 0.001, *n* = 64 blocks).

To elucidate the causal relationship between force modulation and the S1→M1 interaction, we designed a control group of weak (W) stimulus, which aims to elicit a minimal M1 response. Eight sessions of data with stimuli C and W were collected. First, we verified the capacity of stimuli in modulating M1 neural response. The SVM decoder was used to classify trials with and without stimulation. The accuracy was defined as the index of neural modulation (I_N_). It was found that stimulus C exerted significantly greater neural modulations than stimulus W (mean I_N_ for C: 0.68; mean I_N_ for W: 0.55; *p* < 0.01; **Figure** **8**C). Furthermore, we tested whether stimulus C, which evoked greater M1 activity, induced greater force changes than stimulus W. Among the 16 blocks of stimulus C, grip force in 8 blocks (50%) was significantly affected by ICMS, while only 2 (12.5%) blocks of stimulus W revealed significant effects on force (Figure 8A,B; Figure S9, Supporting Information). The deviation of grip force (quantified by I_F_, see Experimental Section) was greater under stimulus C compared to stimulus W (mean I_F_ for C: 0.077; mean I_F_ for W: 0.027 *p* < 0.05, Figure 8D). Additionally, we examined whether neural adaptation attenuated the effects of ICMS on actual grip force. Stimulation at site C consistently induced a stable bias in grip force throughout the 30 trials, indicating that the stimulation effects on actual grip force were not diminished by short‐term adaptation (Figure S7C, Supporting Information). These results indicated that stimuli intervening in S1→M1 interactions were more likely to influence grasping behaviors.

Finally, Pearson's correlation was used to examine the relationship between the magnitudes of neural and behavioral modulation. Considering the potential temporal delay for neural and behavioral correspondence, we conducted correlation analysis across all samples during −500–1500 ms post‐stimulation for I_N_ and I_F_. Significant correlations were found within 140–500 ms of I_N_ and 200–700 ms of I_F_ (Figure 8E). Results revealed positive correlations of the mean I_N_ (140–500 ms) and I_F_ (200–700 ms) for 32 observations from C/W sessions (*r* = 0.5123, *p* < 0.01), and 64 observations from the combinations of both C/W and C/D sessions (*r* = 0.4092, *p* < 0.001). Notably, after excluding two evident outliers, 30 observations from C/D sessions also revealed a significant correlation (*r* = 0.4541, *p* < 0.05, Figure 8F). In summary, stimulations at spatially designed S1 sites could interfere with information transfer between S1 and M1, thereby adjusting sensorimotor coordination during grasping, leading to spontaneous behavior modulation without prior training.

## 3 Discussion

The current study aimed to explore the role of S1→M1 communication in the transfer of tactile information and to assess the potential for regulating sensorimotor coordination by targeting this communication pathway. Our findings revealed the temporal dynamics of tactile neural representations during grasping in S1 and M1, as well as the functional connectivity conveying haptic information between these regions. Through ICMS delivered to S1, we demonstrated a combined effect of stimulation and tactile conditions on M1 responses, suggesting that ICMS designed according to S1→M1 communication can directionally regulate neural encoding and decoding in M1. Finally, the designed ICMS achieved training‐free modulation in actual grip force control.

### 3.1 Neural Representation for Tactile Sensory in S1 and M1 During Grasping

While considerable research examined neural activity in the motor cortex of macaques during reach and grasp tasks,^[^ ^51^, ^54^ ^]^ our study represents the first effort to investigate the influence of textures and loads on the grasping process in both M1 and S1. Our findings indicate that the latency of texture perception is predominantly concentrated at the time of touching the object's surface, whereas the latency of loads subsequently occurs during the phases of grasping and holding. The temporal variation can be reasoned by that neurons receiving input from cutaneous afferents are rapidly activated upon touching,^[^ ^15^, ^17^ ^]^ while those functioning proprioception reflect the continuous force exertion by the upper arm muscles during holding. Additionally, we observed that S1 units exhibited relatively independent functions and locked latencies of textures and loads compared to units in M1, suggesting that S1 responses are linked more directly to external stimuli. In contrast, the dispersed latencies and interacted functions of M1 units implied that M1 activities are indirectly tuned by compiled tactile information from multiple sources, including S1.^[^ ^37^, ^55^ ^]^ Overall, our findings contribute to deconstructing complex neural mechanisms underlying integrated sensorimotor functions during grasping.

### 3.2 Tactile Information Transfer between S1 and M1

Although bidirectional functional connectivity between S1 and M1 was observed in primates,^[^ ^35^, ^56^, ^58^ ^]^ the current study is the first to examine the effects of textures and loads on the interaction between S1 and M1 in grasping. Granger Causality analysis revealed that information transfer from M1 to S1 occurred primarily in the early stages of reaching and grasping, revealing a motor‐dominant characteristic. Such M1→S1 communication may convey the imminent activity of forelimb muscles,^[^ ^39^ ^]^ or trigger the activation of hand‐related sensory neurons to enhance sensory discrimination.^[^ ^40^ ^]^ In contrast, the connectivity from S1 to M1 displayed a bimodal pattern peaking at both stages of early grasping and late holding, conveying information of tactile properties of the target so that M1 could function in maintaining appropriate grip force. Although studies in rodents suggest that proprioception is more inclined to be projected to M1,^[^ ^36^ ^]^ we found that during grasping, the information flow from S1 to M1 was affected by both cutaneous (texture) and proprioceptive (load) stimuli, with the texture effect being more prominent. Our results suggest that the S1→M1 connection conveys information of both texture and load conditions in grasping tasks where proprioception is intertwined with cutaneous sensations (e.g., perception of grip force is affected by textures).

### 3.3 The Interaction between Artificial and Natural Evocation in M1

Consistent with previous studies in non‐human primates and humans,^[^ ^43^, ^59^ ^]^ a significant proportion of M1 units exhibited phase‐locked responses with short latencies of 3–6 ms following S1 stimulation, suggesting a direct S1→M1 connection. Although previous studies identified projections between neurons in the sensorimotor cortex with micro‐stimulation,^[^ ^60^, ^64^ ^]^ few addressed the relationship between neuronal links and behaviors, in particular, behavioral tasks involving sensorimotor coordination. For the first time we found a significant interaction between S1→M1 evocation and tactile conditions on M1 responses during grasping, suggesting a shared pathway for both natural and artificial sensory propagation. The findings shed light on the design of biomimetic stimulation to simulate natural haptic perceptions.

### 3.4 Spontaneous and Rapid Modulation of Grasping via the S1→M1 Pathway

After confirming the S1→M1 pathway for tactile stream, we discovered a biomimetic approach for modulating M1 neural representations and grasping behaviors by encoding ICMS in S1. We achieved directional regulation of the tactile representation at population level by identifying a subset of tactile‐sensitive M1 channels and selecting S1 sites that could evoke neural responses targeting these channels. With designed stimulus pairs corresponding to levels of textures or loads, we were able to either distinguish or confuse the neural representations of tactile levels at will. Our results suggest that natural sensory perception is likely acquired by simulating natural responses in downstream brain regions with upstream stimulations. Previous studies reported that the thalamus→S1 pathway could be used to mimic neural responses in S1 resembling natural sensory input.^[^ ^46^, ^48^, ^65^ ^]^ To the best of our knowledge, the current study is the first to regulate grasping by modulating the pathway between S1 and M1.

While previous research on non‐human primates simulated tactile sensation only on neural activity,^[^ ^46^ ^]^ the current research uncovered the role of S1→M1 interactions in regulating motor behaviors. Our results demonstrate that the biomimetic stimulation based on the S1→M1 interaction leads to spontaneous grip force modulation during grasping and holding. In contrast to approaches that required primates to establish an association between stimulus and motion through learning,^[^ ^2^, ^3^, ^66^ ^]^ the proposed method attempts to achieve spontaneous grip for modulation without training in advance, thereby enabling efficient regulation of sensorimotor coordination. Our findings shed light on the development of natural feedback for neuroprosthetic control in bidirectional BMI.

### 3.5 Benefiting Real‐time Grip Force Control of Robotic Hands

While evocation in M1 by ICMS in S1 was found to compromise motor decoding in bidirectional BMI,^[^ ^43^ ^]^ the current study aims to repurpose the contamination to assist in real‐time control of robotic hands. The designed stimulus D was found to amplify the gap of decoded force across tactile levels, implying that our approach is able to help operators optimize force control of neuroprotheses unconsciously. In real‐world scenarios of robotic hand control, the operator might fail to actively adjust grip force according to tactile feedback, resulting in a grip force that is unresponsive to the target's tactile properties. With the proposed approach, the intervention of ICMS in M1 would shift the decoded grip force toward alignment with the tactile attributes of the target, enabling the robotic hand to deliver optimal grip force. In conclusion, ICMS encoded via the S1→M1 stream offers a promising strategy to reduce operator effort and enhance control efficiency through tactile feedback for bidirectional BMI.

## 4 Experimental Section

### Animals

Two macaques (Macaca Mulatta, male, 4–5 years, 3.9–5 kg) participated in the study. The animals were kept in individual cages with constant temperature and humidity, and were fed by professional caretakers. The experiments were conducted 4 to 5 days per week, during which the water intake was restricted and was used as reward after the animal completed the task. The minimum water intake met the standards for health maintenance. The monkeys had free access to water on weekends. Ethics for the animal experiments was approved by Beijing Institute of Basic Medical Sciences (IACUC‐DWZX‐2024‐554). All experimental procedures were also performed in accordance with the US National Institutes of Health Guide for the Care and Use of Laboratory Animals.

### Behavioral Tasks

The monkeys were trained to complete a grasping task in the dark. They were cued by LED to press a start button and then reached out for a cylindrical target located 40 cm in the front. After the target was grasped, the animals were cued to pull the target 5 cm toward them and hold the target for at least 700 ms to get rewarded. At the end of the trial, the target was released and reset automatically. The minimum inter‐trial interval was 1500 ms. The animals were trained for 6–8 months to complete the experimental procedure. After training, the monkeys were able to complete at least 300 trials within 2 hours per day.

### Implantation

After the monkeys were well‐trained for the experiment, four 32‐channel floating multi‐electrode arrays (FMA, Microprobes, USA) were implanted into the contralateral sensorimotor cortex of their dominant hand. The electrodes were made of iridium, and were 1.5 mm in length. For each animal, one of these arrays was implanted into the primary motor cortex (M1), two were implanted into the primary sensory cortex (S1) and one into the ventral part of the posterior parietal cortex (PPC) brain area (Not described in the current study). The M1 implantation was located against the arcuate sulcus, where had been reported to represent hand and upper limb movement. The S1 implantation was close to the central sulcus, with the electrodes mostly located in Brodmann Area 1. The PPC implantation was positioned in PF, which was believed to be associated with grasping and tactile sensing.

### Neurophysiological Recording and Preprocessing

Neural activity was recorded by an OmniPlex neurophysiological acquisition system (Plexon, USA) with a sampling frequency of 40 kHz. The wide‐band data was filtered by a 300 Hz Bessel high‐pass filter. Subsequently, spike waveforms were detected with a threshold of −4.5 RMS. Finally, the detected spike waveforms were manually sorted into units, which were assumed to reflect firing of neurons.

For data involving intracortical micro‐stimulation, the high‐pass filter was set to 750 Hz due to more low‐frequency artifacts induced by electrical stimulation. The threshold of waveform detection varied from −1.5 to −4.5 RMS depending on the magnitude of artifacts. Multi‐unit data (numbered as channels) was directly used for analysis after threshold crossing without manual sorting. The preprocessing was performed with Plexon Offline Sorter.

To quantify grasping behaviors, real‐time grip force was also collected by a force dynamometer, which was used as the cylindrical target in our paradigm. The grip force was recorded simultaneously with neural data by an analog channel of OmniPlex.

### Intracortical Micro‐Stimulation

ICMS was delivered by a PlexStim electrical stimulator (Plexon, USA). Stimulation was designed as trains of charge‐balanced, current‐controlled square‐wave pulses. Each pulse consisted of a cathodal phase of 250µs, an interphase interval of 50 µs, and an anodal phase of 250 µs. The amplitude of stimulation ranged from 20 to 80 µA, and the frequency ranged from 5 to 100 Hz.

### Tactile Stimuli

Levels of textures and loads of the target for grasping were taken as experimental variables. The surface of the dynamometer was coated with various materials, allowing for the setting of surface texture. To select suitable texture materials, we tested static friction coefficients toward leather (as an alternative to hand skin) for more than ten types of textures such as sandpaper, silk, and wool. Ultimately, four texture materials were chosen for the experiment: waterproof fabric (WB), P7000 sandpaper, P400 sandpaper, and P36 sandpaper (static friction coefficients: 0.371, 0.686, 1.225, and 1.986, respectively). The load for the grasping process could be controlled by adjusting the weights hanged at the distal end of the dynamometer. Considering the arm strength of the monkeys, a load range of 400–700 g was selected for the experiment.

### Experimental Procedures:Grasping under Crossed Conditions of Texture & Load

A 3 × 3 experimental design incorporating two independent variables—texture and load—was employed. The texture levels were categorized either as P36/P400/P7000 sandpapers or as waterproof fabric/P400/P7000 sandpapers, while the load levels were set at 400/550/700 g. Crossing these factors produced nine groups, each comprising approximately 30 trials (with a minimum of 25 trials per group), yielding a total of around 270 trials across nine tactile conditions per session. To mitigate potential time and fatigue effects, each session was divided into 18 blocks: the first nine blocks featured unique texture/load combinations, while the remaining nine blocks presented these combinations in reverse order. Data from eight sessions per monkey were analyzed, consisting of four sessions with P36/P400/P7000 textures and four with P400/P7000/WB textures, resulting in 16 datasets included in the analysis.

### Experimental Procedures:Resting‐State ICMS to S1

Monkeys received passive S1 stimulation without engaging in behavioral tasks. Stimulation was delivered via two electrodes in S1, and neural responses were recorded in M1. In each S1 array, 30 channels were stimulated for monkey A and 15 channels for monkey B. To compare M1 responses across stimulation parameters, three amplitude levels were tested (20/50/80 µA at 100 Hz for monkey A; 20/40/60 µA at 50 Hz for monkey B) along with four frequency levels (10/25/50/100 Hz at 80 µA for monkey A; 5/10/25/50 Hz at 60 µA for monkey B). Each stimulation lasted one second with a three‐second interval, and each channel underwent 14 repetitions per parameter set. During stimulation, the monkeys remained seated in a dark environment with their hands restrained. Datasets were excluded if substantial movements occurred during data collection. A total of eight valid datasets were analyzed (2 monkeys * 2 S1 electrodes * amplitude/frequency).

### Experimental Procedures:Simultaneous ICMS Applied during Grasping

Data collection spanned two consecutive days, with the first day's experiment focused on identifying stimulation sites capable of modulating tactile sensation and the second day dedicated to evaluating the effects of ICMS on grasping performance. On the first day, monkeys completed 100 grasping trials under texture (P400/P7000) or load (400/700 g) conditions. To mitigate time and fatigue effects, trials were organized into four blocks, with the final two blocks presented in the reverse order of the initial two (e.g., 400/700/700/400 g). Following these trials, cyclic electrical stimulation was delivered to channels of an S1 array in the resting state, using parameters according to those described above (monkey A: amplitude 80 µA, frequency 100 Hz; monkey B: amplitude 60 µA). Analysis of day one data identified two stimulus pairs (each comprising two stimulation channels corresponding to tactile conditions) that rendered the conditions either distinguishable (D) or confusable (C) for day two (details on identifying C/D pairs are provided in Data Analysis).

On the second day, the ICMS was applied synchronously to selected channels during task performance. The ICMS was triggered by touching the target, with a median delay of 210 ms, aligning the onset of ICMS with the transition from touching to grasping—an optimal window for sensory feedback. The ICMS lasted one second, encompassing the entire grasping phase, with parameters identical to those used on day one. Data were collected in four blocks combining tactile conditions (T1/T2) and stimulation conditions (C/D). Each block contained 60 trials, evenly split between ICMS‐stimulated trials and non‐stimulated controls, presented in a pseudo‐randomized order. Each monkey completed four sessions of data collection (texture/load conditions for two S1 arrays), yielding a total of eight datasets for analysis.

To further explore the S1‐M1 interaction in grip force regulation, a weak (W) stimulus pair eliciting minimal M1 response was also identified (detailed procedures in Data Analysis). The above protocol was repeated with C/W stimuli pairs. Each monkey completed four additional sessions (texture/load conditions for two S1 electrodes), resulting in eight C/W datasets.

### Data Analysis:Mixed Effect of Textrure & Load on Single Unit Activity

The effect size (η^2^) of two‐way ANOVA was used to quantify the influence of textures, loads, and their interaction on the firing rate of units^[^ ^29^ ^]^:

(1) $$\eta_{i}^{2}=\frac{SS_{i}}{SS_{texture}+SS_{load}+SS_{texture\times load}+SS_{error}}$$

where *SS* represents the sum of squares and *i* represents the quantified variable (texture/load/texture × load interaction). To illustrate the temporal effects of tactile conditions, time‐series *η* ^2^ values were calculated using a 200 ms moving window with a 50 ms step. Data from all trials were aligned to the initial touch of the target (0 ms). Units with fewer than five significant responses to both textures and loads within the ‐200–1000 ms range were considered as tactilely insensitive and excluded from further analysis. The corrected significance threshold was set at *p* < 0.0036 (*p* < 0.05, adjusted for 3 factors * 23 total time points / 5 inclusion trials).

### Data Analysis:Population Classification of Tactile Conditions

An SVM classifier with a Gaussian kernel was constructed to characterize population‐level representations of tactile conditions in S1 and M1. Classification accuracy over time was evaluated using a 200 ms moving window with a 50 ms step. The input data for the classifier were segmented into 4 bins (50 ms per bin). Textures and loads were decoded independently. Accuracy was assessed through 10‐fold cross‐validation.

To investigate the impact of unit counts on decoding performance, we altered the proportion of input units in M1 and S1. The units were dropped in the order of η^2^ for textures/loads. Also, a random‐order test was repeated for 10 times to form a mean accuracy curve. Given the variability in tactile effects between the Touching (T) stage (−50–150 ms) and the Grasping & Holding (GH) stage (350–550 ms), classification was performed separately for these two intervals.

### Data Analysis:Conditional Granger Causality

A non‐parametric Granger causality analysis was performed on spike trains to assess the directional information transfer between units in S1 and M1.^[^ ^49^, ^67^ ^]^ Conditional Granger causality (CGC) could evaluate the functional connectivity between two units (i and j) after accounting for the influence from other units (k). The conditional GC in the frequency domain from unit j to unit i, conditional on k, can be represented as:

(2) $$I_{j\to i|k}\left(f\right)=\ln\frac{\Sigma_{ii}\left(i,k\right)}{\left|{\tilde{H}}_{ii}\left(f\right){\tilde{\Sigma }}_{ii}\left(i,j,k\right){\tilde{H}}_{ii}^{*}\left(f\right)\right|}$$

where Σ_*ii*_(*i*,*k*) represents the residuals of unit i that are not explained by the joint regression of i and k, ${\tilde{\Sigma }}_{ii}\left(i,j,k\right)$ is the noise covariance matrix in the joint regression of i, j, k, and ${\tilde{H}}_{ii}\left(f\right)$ is the normalized transfer function. The asterisk denotes the complex conjugate.

Given the overlapping functions between S1 and M1, we computed the S1→M1 Granger Causality (GC) while excluding the influence of other M1 units (S1→M1|M1) and, conversely, calculated the M1→S1 GC while excluding the influence of other S1 units (M1→S1|S1). To prevent issues related to singular matrices caused by low firing rates, only the top 10 firing rate units from S1 and M1 in each dataset were selected for continuous CGC analysis (100 unit pairs for each dataset, 1600 pairs in total). A moving window of 300 ms with a 50 ms step was applied to data within the ‐500–1000 ms range. CGC analysis was conducted across nine tactile conditions (3 textures × 3 loads). The statistical significance of the CGC was assessed using the spike jitter method.^[^ ^50^ ^]^ Spikes were randomly jittered within a ±10 ms range, and CGCs were recalculated using surrogate data. This procedure was repeated 30 times to generate a distribution of surrogate CGC values. If the original mean CGC value exceeded the 95th percentile of the surrogate distribution, it was considered statistically significant. The following analysis was applied for all 1600 unit‐pairs, as well as for pairs with significant CGCs only (923 pairs for M1→S1|S1 and 659 pairs for S1→M1|M1).

To characterize the differences of CGCs across varying tactile conditions, we conducted a resampling of trials, selecting 20% of the trials with replacement for 20 iterations, yielding a distribution of CGC values. For each resampling instance, CGC was computed during stages T (−100–200 ms) and GH (300–600 ms). We then employed a two‐way ANOVA to assess the significance of the CGC's sensitivity to texture and load conditions.

Principal Component Analysis (PCA) was applied for dimensionality reduction of continuous CGCs. CGCs under all nine tactile conditions were concatenated for PCA. The principal components (PCs) were ranked by explained variances. As effects of textures and loads appeared in PC3 and PC4, resampled data were projected onto PC3 and PC4 to obtain the distribution of samples within the PC3/PC4 projection plane.

### Data Analysis:M1 Response during Resting‐state ICMS in S1

To validate the modulation of S1 ICMS on M1 response, pulse‐related neural activity with baseline was compared. Data was aligned to the onset of the pulse. As the maximum of frequency was 100 Hz in the experiment, only the first 10 ms after pulse were taken into analysis to facilitate cross‐frequency comparisons. To eliminate stimulation artifacts, the first 3 ms after each pulse and the final 1 ms before the subsequent pulse were excluded. Thus, the firing rate from 3 to 9 ms post‐pulse was compared to baseline activity, defined as the firing rate during 3–9 ms following a sham stimulation (0 amplitude). An M1 channel was considered activated by S1 stimulation if its pulse‐related firing rate significantly exceeded the baseline (*p* < 0.01). Conversely, a significantly lower pulse‐related response indicated an inhibitory effect on the M1 channel. M1 channels with a normalized firing rate below 2 Hz were excluded from excitatory classification to prevent misclassification due to incidental activity.

The pulse‐triggered average (PTA) was calculated to assess whether M1 channel excitation was phase‐locked to the pulse.^[^ ^43^ ^]^ Spike trains within the inter‐pulse interval were divided into 0.5 ms bins, and spike probabilities were calculated for each bin. To determine the presence of a significant phase‐locked response, the bin with the highest spike probability and its two adjacent bins were averaged. The median spike probability across all bins during the inter‐pulse interval was also calculated to quantify non‐phase‐locked responses. The difference between these values constituted the phase‐locking index. For each M1 response triggered by S1 stimulation, 20% of PTAs were sampled and shuffled to create a PTA with a matched spike count. This process was repeated 1000 times to generate a null distribution for phase locking. If the phase‐locking index exceeded the 99th percentile of the surrogate data (p < 0.01), the PTA was deemed to exhibit significant phase locking.

We categorized the responses of M1 into three types: phase‐locked excitation, non‐phase‐locked excitation, and inhibition. To investigate the effects of stimulation parameters on M1 responses, we compared the proportions of these three types of responses under different amplitudes and frequencies. Additionally, we selected stimulation and response channels that could produce either type of response under at least one level of stimulation, and compared the post‐pulse firing probability of these channels across various parameters (Figure 4C,D; Figure S3A,B, Supporting Information).

### Data Analysis:Stimulation Site Selection

First, M1 channels sensitive to tactile conditions were identified from data collected on Day 1, Experiment 3. Data was aligned to the initial hand‐target contact, and an SVM classifier was applied to 50 ms binned data (0–500 ms) from each M1 channel to classify tactile conditions. M1 channels were ranked by classification accuracy, assessed via 10‐fold cross‐validation. Peristimulus time histograms (PSTHs) were plotted to visually inspect firing rate differences across tactile conditions. Channels exhibiting high classification accuracy and clear firing rate distinctions between conditions were considered tactilely sensitive.

Secondly, S1 sites capable of modulating M1 responses were identified using rest‐state data with ICMS. Firing rates between 3–9 ms post‐pulse were averaged and compared to those following sham stimulation. S1 sites that produced significant excitatory or inhibitory effects (*p* < 0.01) on M1 channels sensitive to tactile conditions were selected for further analysis.

Finally, we manually selected S1 sites that tend to drive the neural representations in M1 to be either distinguishable (D) or confusable (C) between the two tactile conditions (Figure 6A). Assuming that an M1 channel exhibited a significantly different firing rate between the two tactile conditions (for example, T1 > T2), and was elicited by stimulation from site A in S1. The firing rate of the channel under condition T1 (FR_T1_) might be further increased by superimposing stimulus A simultaneously, amplifying the disparity between FR_T1_ and FR_T2_. Conversely, stimulus A might increase FR_T2_, narrowing the gap between FR_T1_ and FR_T2_. Thus, stimulus A could be a distinguishable (D) stimulus under T1, but a confusable (C) stimulus under T2, as the effects on the firing rate gap are opposite between tactile conditions. The selected pairs of C and D stimuli were based on the same two sites but corresponded to opposite tactile conditions (Pair D: T1‐stim A, T2‐stim B; Pair C: T1‐stim B, T2‐stim A). According to the above design, the principles for selecting stimulation pairs are as follows: (1) Pair D stimuli should increase the gap of firing rate in as many M1 tactile‐sensitive channels as possible, while Pair C stimuli should reduce the gap in as many M1 tactile‐sensitive channels as possible; (2) Since one S1 stimulus may have heterogeneous effects on multiple M1 channels, stimulation sites that simultaneously increase and decrease tactile gaps in different M1 channels should be avoided.

In addition to pairs C and D, we also designed weak stimulus (W), which led to minimum M1 response. The artifacts in M1 caused by stimulus W were as obvious as those of C and D, so the weak effect of stimulus W was not due to bad S1 sites.

### Data Analysis:Conditional Granger Causality with ICMS data

To investigate the effect of ICMS on CGC between S1 and M1, we performed CGC analysis on grasping datasets collected under ICMS stimulation. This analysis was based on 8 grasping‐with‐ICMS datasets. To ensure that the channels included had sufficiently high firing rates to support the computation of non‐parametric CGC, only the top 10 channels with the highest firing rates were selected for each dataset (with one dataset including only 9 channels per region due to limited firing rates). As a result, a total of 781 channel pairs were included in the analysis (see Section 4.8.3 for details of CGC computation).

### Data Analysis:Interaction between ICMS and Tactile Conditions

To investigate whether tactile and ICMS stimulation shares a common neural projection mechanism, we examined their interactive effects on neuronal activity. Two‐way ANOVA was conducted to assess the interaction between ICMS pairs and tactile conditions on the average firing rate within 0–400 ms after stimulation onset, a period during which M1 firing rates are modulated by both ICMS and tactile stimuli. Channels with significant interaction were filtered by *p* < 0.05.

If an interaction between ICMS and tactile stimulation was observed in a channel, the response evoked by ICMS should vary across tactile conditions. To test this, we trained an SVM classifier to distinguish the two stimulus sites. Only single‐channel data from channels showing significant interactions were included. For each tactile condition (T1/T2), the data was split into training (80%) and test (20%) sets. The model was trained on the T1 training set and tested on both T1 and T2 test sets. Interaction was indicated if the T1 model significantly outperformed on the T1 test set compared to T2. The classification accuracy was assessed using 5‐fold cross‐validation, with a 200 ms moving window (step size 40 ms) to examine the accuracy as a function of time. To assess whether the interaction was present across the neural population, we applied this classification procedure to all M1 channels in each dataset.

### Data Analysis:Effect of ICMS on Neural Representation

To evaluate the effects of stimuli C and D on tactile representation in M1, an SVM decoder with a Gaussian kernel was built to classify tactile conditions continuously with single‐channel firing rates. We included all channels that exhibited significant main effects of texture and load (*p* < 0.05) in the prior ANOVA, as channels unresponsive to tactile input would not contribute meaningfully to texture classification. The grasping data collected on day 1 was taken as the training set, and the data collected on day 2 synchronizing grasping and ICMS served as the testing set. The data were divided into 40 ms bins. Classification was performed on a moving window of 5 bins (200 ms) with a step of 1 bin. For the consistency between the training and testing sets, the first 3 and the last 1 milliseconds in every 10 milliseconds were blanked as artifacts for both training and testing sets. To compare the differences between stimulated and non‐stimulated conditions, we defined the index of difference as:

(3) $$I_{ACC}\left(t\right)=\frac{ACC_{ICMS}\left(t\right)-ACC_{NONE}\left(t\right)}{ACC_{NONE}\left(t\right)}$$

where *ACC* represents the classification accuracy for tactile conditions. Additionally, we also verified the effects of stimuli C and D on neural populations. Instead of single M1 channel, all M1 channels in each dataset served as inputs for the classifier. In particular, we defined the Grasping stage (0–280 ms after stimulation) and the Holding stage (400–680 ms after stimulation) to evaluate the effects of ICMS on tactile representation across stages of motion.

### Data Analysis:Effects of ICMS on Grip Force Decoding

A Support Vector Regression (SVR) model with a linear kernel was built to fit the grip force by the population neural response from M1. Similar to the procedure for SVM classification, spike trains were divided into bins of 40 ms and a moving window of 5 bins (200 ms) was used as the input of the regression model, with a step of 40 ms. The grasping data collected on day 1 was taken as the training set while the data synchronizing grasping and ICMS on day 2 served as the testing set.

We defined the bias between the decoded and actual grip force as:

(4) $$Bias\left(t\right)=\frac{F_{Decoded}\left(t\right)-F_{Real}\left(t\right)}{F_{Real}\left(t\right)}$$

where *F* is the real‐time grip force. Further, the index of decoding bias between trials with and without ICMS was defined as the normalized difference of *Bias*(*t*).

### Data Analysis:Effects of ICMS on Actual Grip Force

By visual inspection, differences in grip force caused by ICMS were observed 300–700 ms after stimulation (Figure S8, Supporting Information). Accordingly, the average grip force between the stimulated and non‐stimulated trials during 300–700 ms was compared.

To verify the role of the S1‐M1 interaction in the modulation of grip force, datasets containing the C and W stimuli were analyzed. First, an SVM classifier was used to classify trials with or without ICMS. Accuracy was used to index the neural variation caused by ICMS (I_N_), where a higher accuracy indicated a stronger intervention on M1 response. A 200 ms moving window with a step of 40 ms was used for continuous classification. Next, we defined the index of difference between ICMS and Non‐ICMS trials for grip force as:

(5) $$I_{F}\left(t\right)=\frac{F_{ICMS}\left(t\right)-F_{NONE}\left(t\right)}{F_{NONE}\left(t\right)}$$

Consistently, the 200 ms moving window was applied for index *F*, where *F(t)* was the average grip force for each window. To further validate the relationship between S1‐M1 information transfer and grip force regulation, we conducted a correlation analysis between the classification accuracy and the grip force difference coefficient. Considering the potential temporal delay between neural representation and behavior, we performed correlation analysis on all time points of I_N_ and I_F_ within ‐500–1500 ms.

### Data Analysis:Statistics

Unless specified, two‐way ANOVA was used to analyze the main effects of independent variables (e.g., texture/load or tactile condition/stimulus condition) and their interaction on neural responses across multiple trials, with Bonferroni correction applied for post‐hoc comparisons. Correlations were evaluated using Pearson's r. Non‐parametric methods were employed for comparisons between two experimental conditions due to small sample sizes in some tests. The Wilcoxon Signed‐Rank Test was used for paired data, while the Wilcoxon Rank‐Sum Test was used otherwise. One‐sided tests were used for those comparisons with clear hypothesis (e.g., the stimulus designed to increase firing rates was expected to cause greater response), otherwise two‐sided tests were conducted. All the statistical analyses were conducted in MATLAB 2020b (MathWorks, USA).

## Conflict of Interest

The authors declare no conflict of interest.

## Author Contributions

Q.Z., C.W., and J.Z. conceptualized the project; Q.Z. and S.L. designed and carried out the experiments; Q.Z., Q.Z., and Y.Z. performed the surgeries; Q.Z., B.L., and X.Y. performed the data analysis; Q.Z. wrote the original draft; Q.Z., Z.W., and J.Z. reviewed and edited the final manuscript.

## Supporting information



Supporting Information

## Data Availability

The data that support the findings of this study are available from the corresponding author upon reasonable request.
