# Supplementary material for: Training‐Free Regulation of Grasping by Intracortical Tactile Feedback Designed via S1‐M1 Communication
Source: Adv Sci (Weinh). 2025 Jun 30;12(36):e03011. doi: 10.1002/advs.202503011 (PMC12462993; doi:10.1002/advs.202503011)
Supplement: Supplementary file 1 — Supporting Information [file ADVS-12-e03011-s001.docx]

**Supplementary Information**

**Training-free Regulation of Grasping by Intracortical Tactile Feedback Designed via S1-M1 Communication**

*Qi Zhang, Bing Liu, Zhemeng Wang, Jiayue Zhou, Xingyi Yang, Qian Zhou, Yuwei Zhao, Siwei Li, Jin Zhou^*^, Changyong Wang^*^*


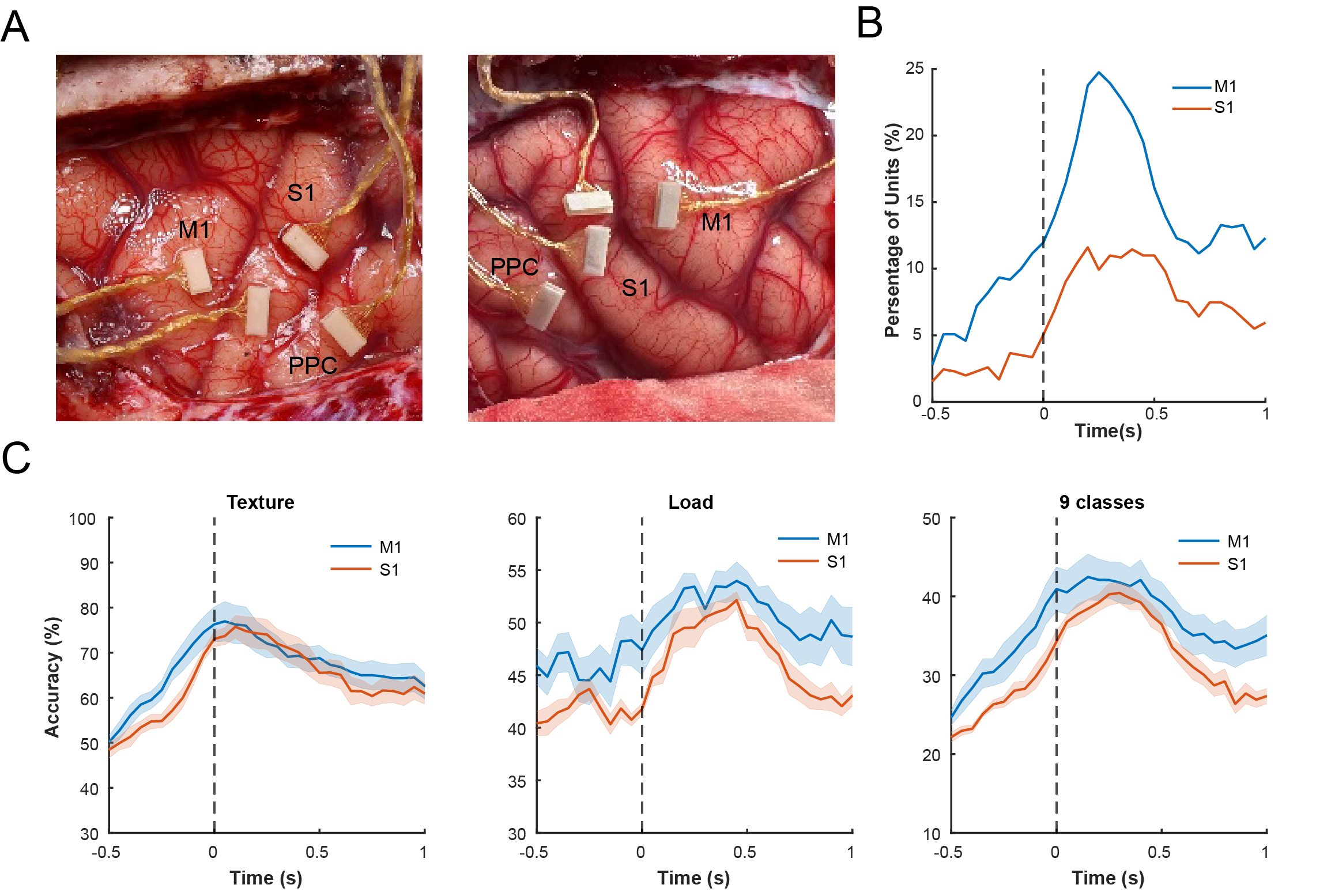


**Figure S1. Neural Representation of Texture and Load in S1 and M1, Related to Figure 2.** **(A)** Implantations in the sensorimotor cortex. Electrodes for monkey A were implanted in the left hemisphere, while those for monkey B were implanted in the right hemisphere. Each monkey received one electrode in M1, two in S1, and one in the PPC (not discussed in this study). **(B)** Proportions of units reacting to both texture and load as functions of time. M1 achieves a greater proportion of units to respond to both texture and load conditions than S1. **(C)** Classification of texture (left), load (middle), and texture*load (right) by firing rates of all units in a session. The classification for texture achieved peaked at around stage T, while that for load peaked at stage GH. The shaded areas represent the standard deviation obtained through bootstrap sampling (n=16).


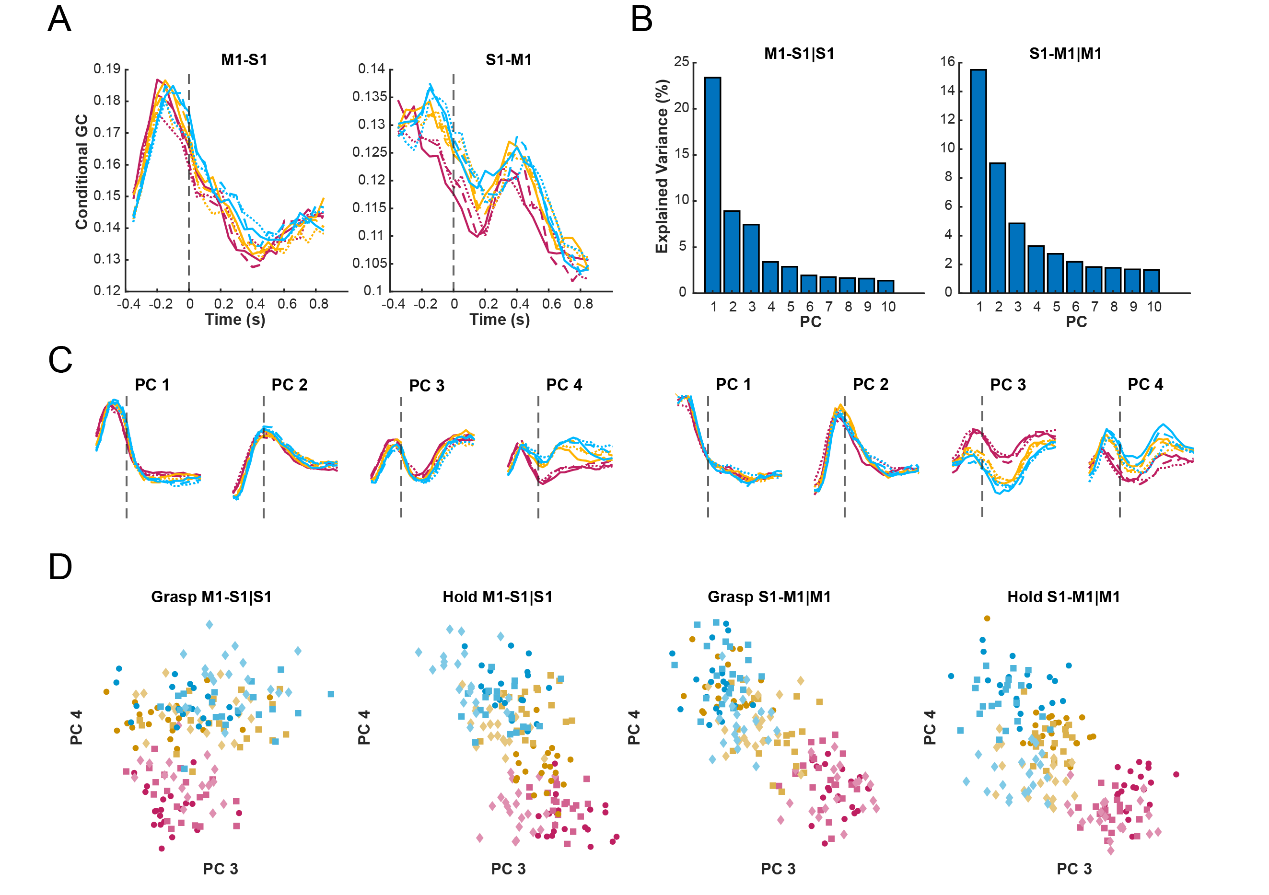


**Figure S2: Conditional Granger Causality for All Unit Pairs, Related to Figure 3.** **(A)** Mean Conditional Granger Causality (CGC) for 10*10 unit pairs in all 16 sessions (Including non-significant pairs). CGCs from M1 to S1 conditional on S1 (M1→S1|S1) peak at reaching before touching the target (left). CGCs from S1 to M1 conditional on M1 (S1-M1|M1) show two peaks during reaching and grasping (right). The colors represent textures of varying roughness, with blue indicating the smoothest texture and pink the indicating roughest. **(B)** Variance explained by each PC after PCA for all 1600 unit pairs. A total of 43.16% of the variance for CGC of M1→S1|S1 is collectively explained by the first four PCs, while 32.66% is explained for CGC of S1→M1|M1. **(C)** The first four principal components (PC) after PCA for all 1600 unit pairs. Similar to Figure 3C, S1→M1|M1 CGC shows sensitivity to texture levels from PC3 onwards, and sensitivity to load levels from PC4 onwards. For the M1→S1|S1 CGC, effect of texture begins to manifest from PC4 onwards. **(D)** Projection of CGC on PC3 and PC4 during stage T and GH. The colors represent textures of varying roughness (same with a)), while the shapes of scatters represent levels of load: dots for 400 g, squares for 550 g, and diamonds for 700 g. Each observation reflects CGC from resampled trials (20% trials resampled for 20 times).


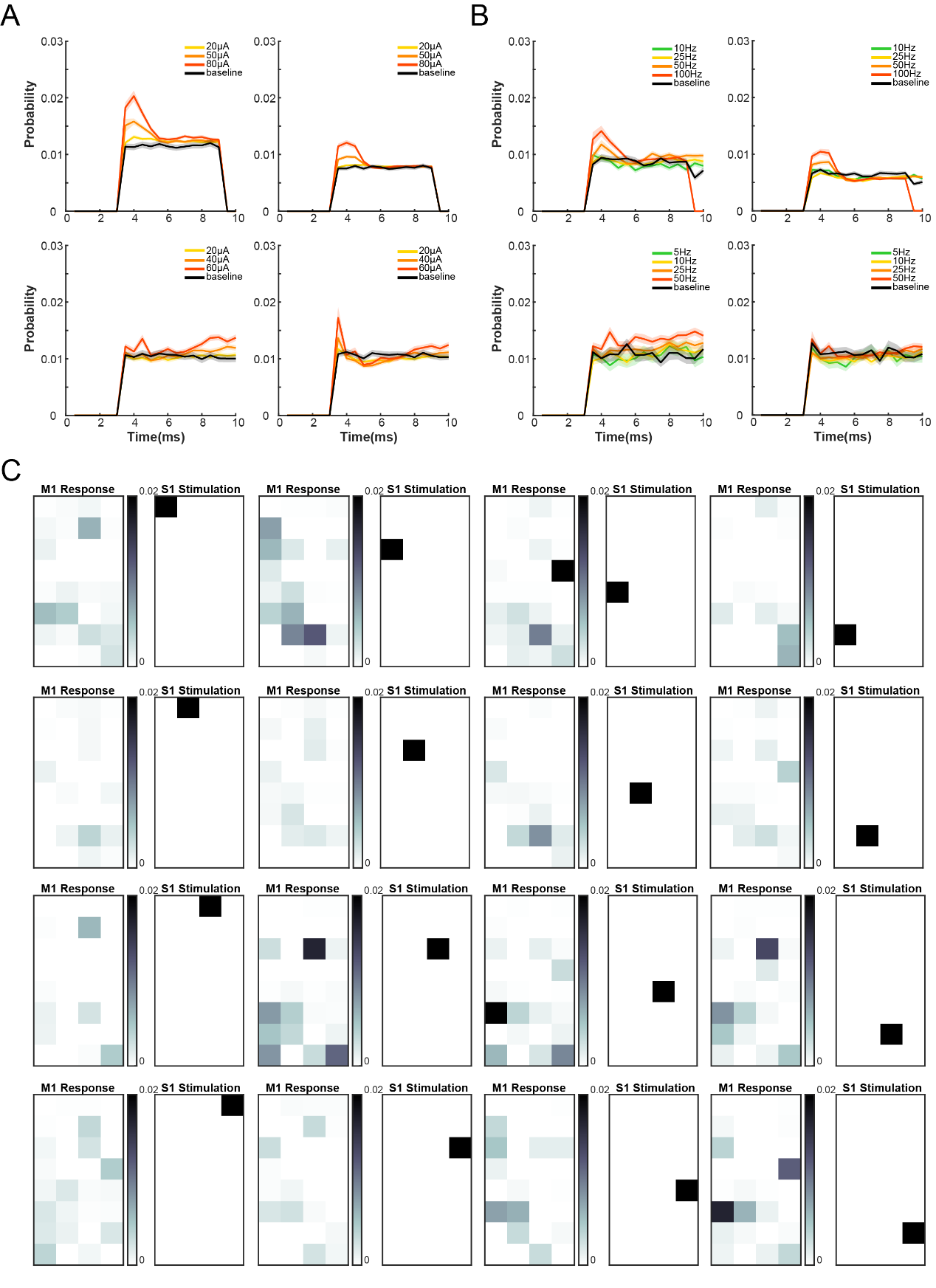


**Figure S3: M1 Responses to S1 Stimulations, Related to Figure 4.** **(A)** Effects of ICMS amplitudes on M1 responses. The response intensity increases with amplitude for non-phase-locked elicitations (left), but is vague for inhibitory responses (right). The top row presents data from monkey A, while the bottom row presents data from monkey B. **(B)** Effects of ICMS frequencies on M1 Responses. Similar to phase-locked elicitations (Figure 4D), greater response intensity is found for frequencies over 50Hz than lower frequencies for non-phase-locked elicitations (left). However, the effect of frequency on inhibitions remains vague (right). The top row presents data from monkey A, while the bottom row presents data from monkey B. **(C)** M1 response corresponding to S1 ICMS across all stimulated sites from a session of monkey B. The spatial pattern of M1 response differs across locations of stimulated sites. Responses are quantified as the difference between the post-stimulation firing rate and the baseline firing rate without stimulation.

**
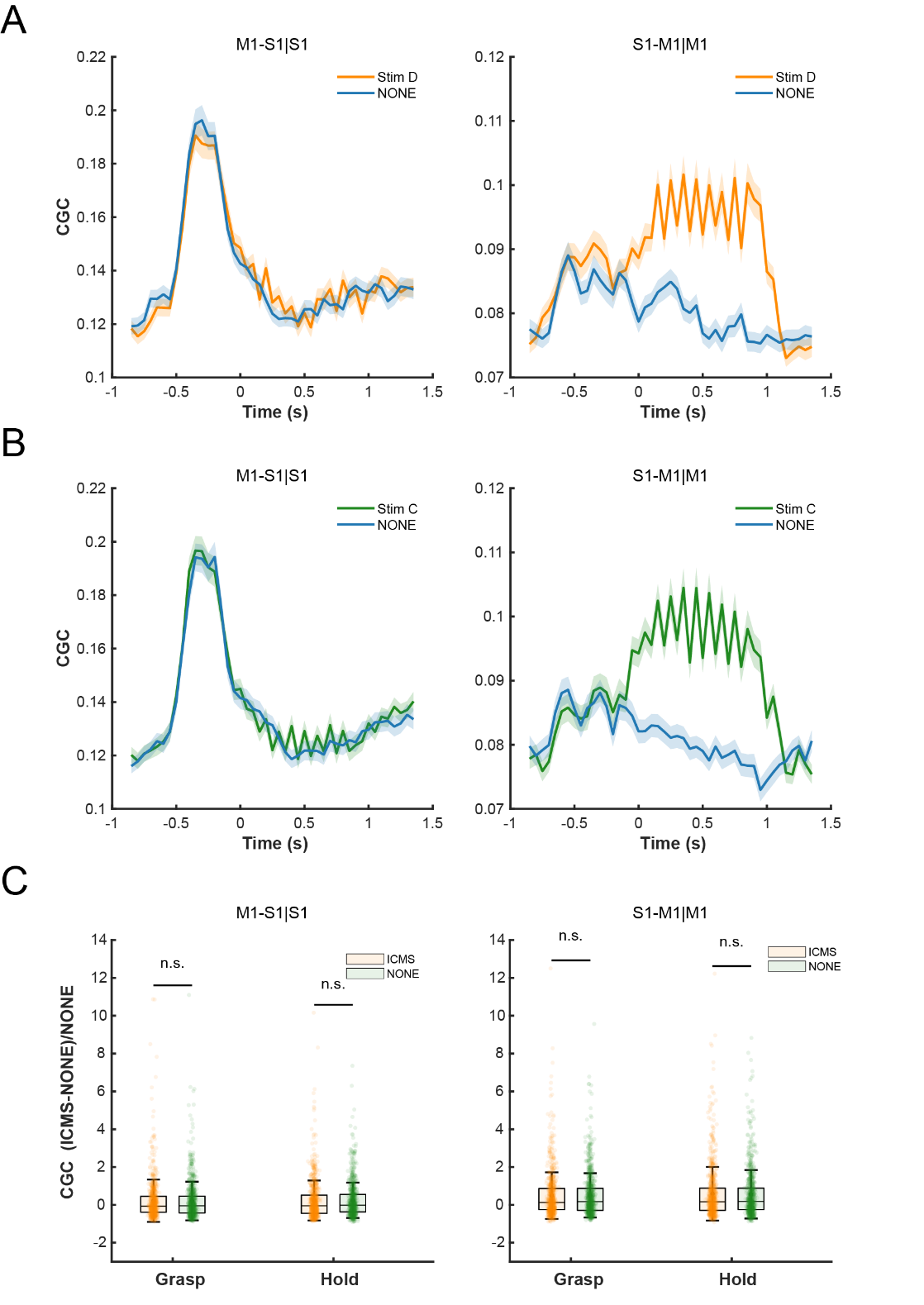
**

**Figure S4. Effects of stimulation site on conditional Granger causality between S1 and M1, related to Figure 5. (A)** Average CGC of all 781 M1–S1 channel pairs over time under stimulation D versus no stimulation. Stimulus D selectively increases CGC S1→M1|M1 (right), with no change in CGC M1→S1|S1 (left). **(B)** Similar to **(A)**, but for stimulus C. Stimulus C similarly enhances CGC S1→M1|M1 (right), without affecting CGC M1→S1|S1 (left). **(C)** Comparison of CGC changes induced by stimuli C and D during grasping and holding. No significant differences are found between C and D in either direction (Wilcoxon signed-rank test, n = 781 channel pairs; n.s.: p > 0.05; *: p < 0.05; **: p < 0.01; ***: p < 0.001).


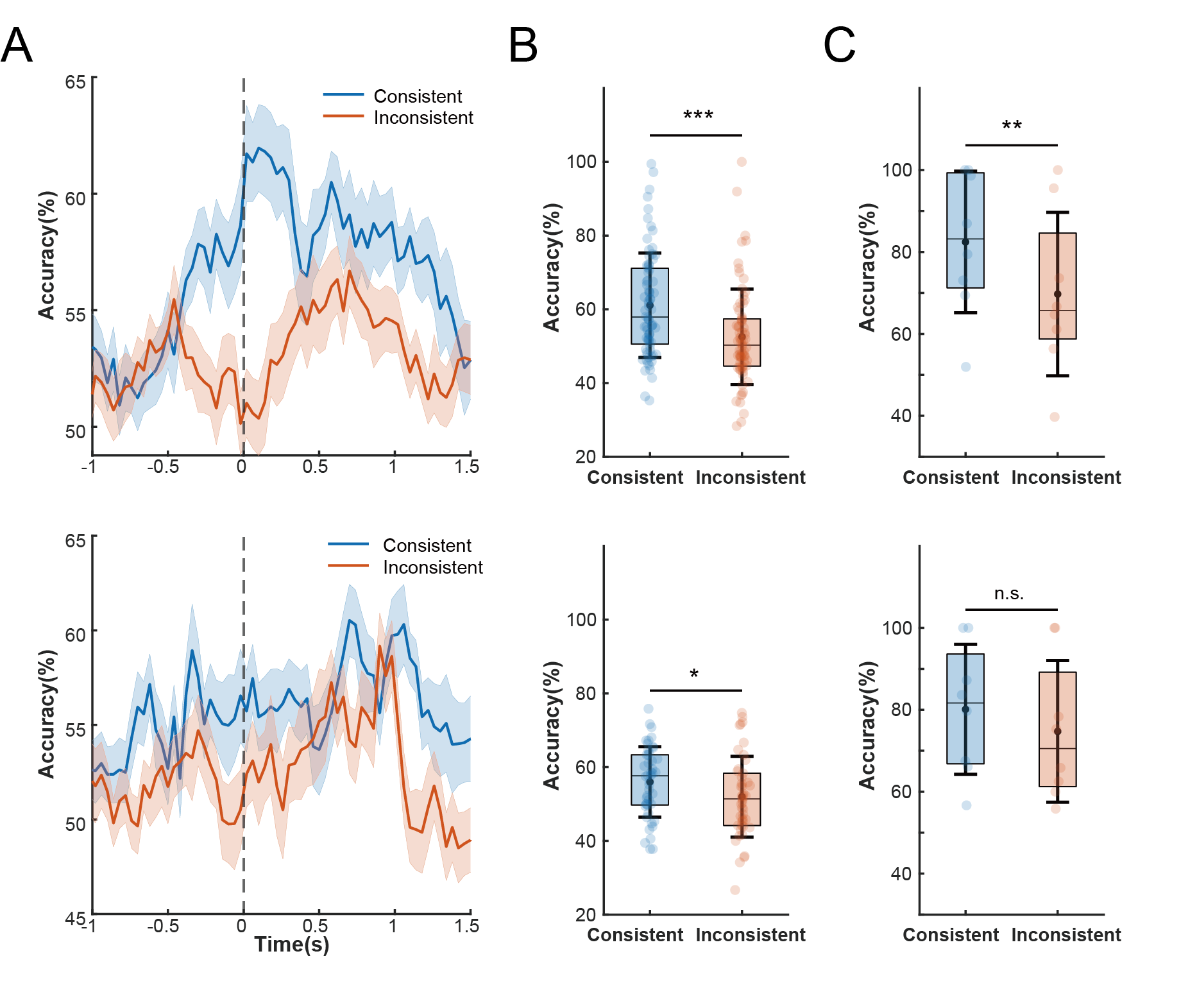


**Figure S5. Tactile-Dependency of ICMS-evoked M1 Response for Conditions of Texture and Load, Related to Figure 5.** (**A**) Performance of classifying stimulation sites by channels sensitive to the interaction between tactile and stimulation conditions. Each observation corresponds to one channel in M1. Accuracies of which training and testing sets are within a consistent tactile level (blue) are greater than those of inconsistent tactile levels (red) for both texture (the upper panel, n=38 channels) and load (the lower panel, n=23 channels). (**B**) Averaged classification accuracy during 0~400 ms. Similar to (A), the accuracies of consistent training and testing sets are greater than those of inconsistent sets for both texture (the upper panel, p<0.001, Wilcoxon Signed-rank test, n=38) and load (the lower panel, p<0.05, n=23), suggesting that the evocation of ICMS is tactile-dependent. (**C**) Average classification accuracy for populations of M1 channels during 0~400 ms. The population accuracies of consistent training and testing sets are greater than those of inconsistent sets only in sessions of varied texture levels (the upper pane, p<0.01, n=8 blocks), but not in sessions of varied load levels (the lower panel, p>0.05, n=8 blocks). Each observation represents accuracy from one session. n.s.: p>0.05; *: p<0.05; **: p<0.01; ***: p<0.001.


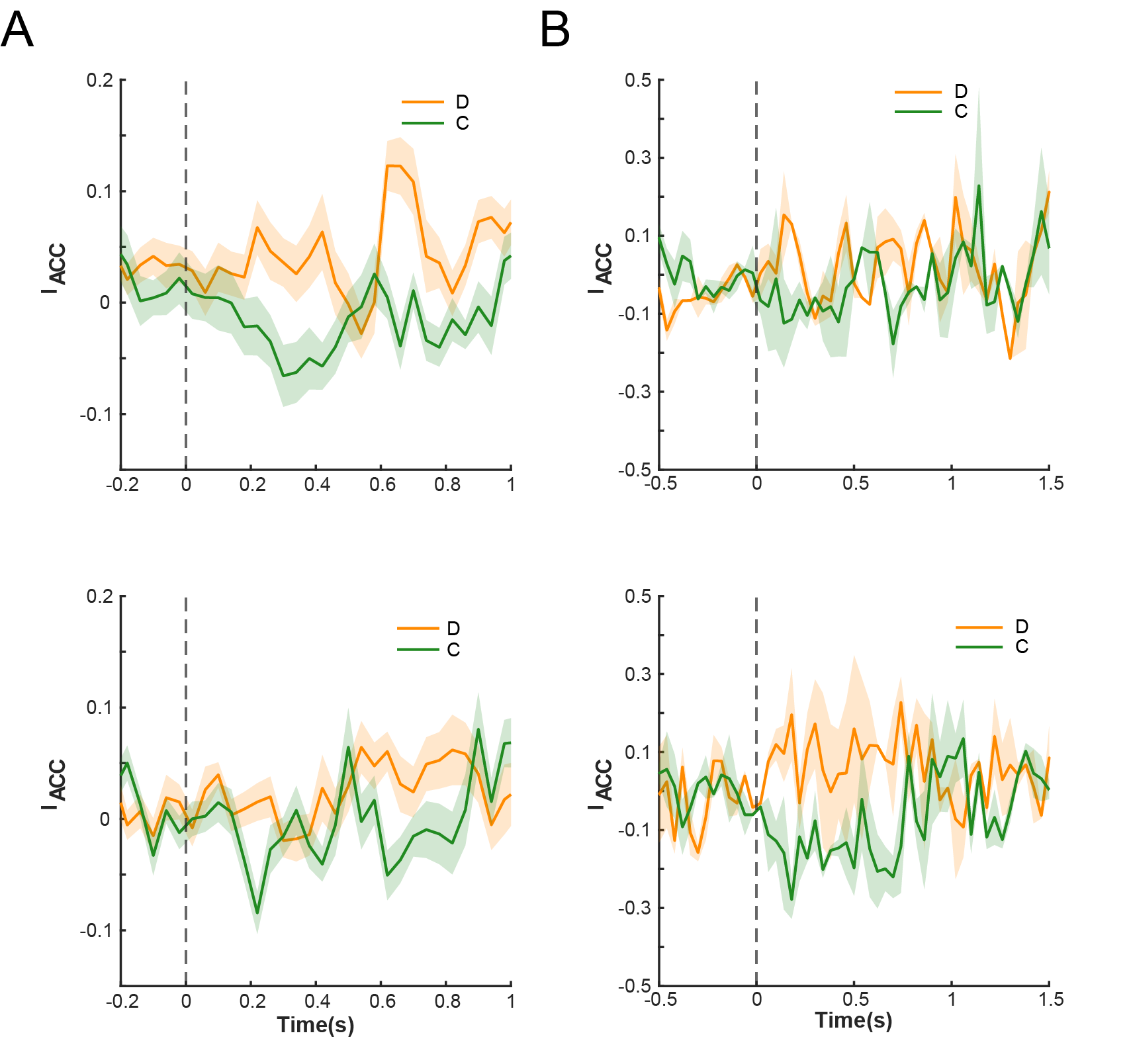


**Figure S6. Modulation of the Designed ICMS on Neural Representation of Texture and Load, Related to Figure 6.** (**A**) Index of difference for single-channel classification (I_ACC_) of tactile levels. Positive effects of stimulus D are shown for sessions of both texture (the upper panel, n=49 channels) and load (the lower panel, n=33 channels) levels. (**B**) Performance of classification for tactile levels by population channels. Accuracies with stimulus D are greater compared to those without stimulation for sessions of both texture (the upper panel) and load (the lower panel), while accuracies with stimulus C decrease after the onset of stimulation. (**C**) I_ACC_ for population classification. Alike (**B**), positive effects of stimulus D, as well as negative effects of stimulus C are shown on levels of load (the lower panel, n=4) over the whole grasp & hold process. Whereas slighter effects appear for levels of texture (the upper panel, n=4) during grasping (0~280 ms).

**
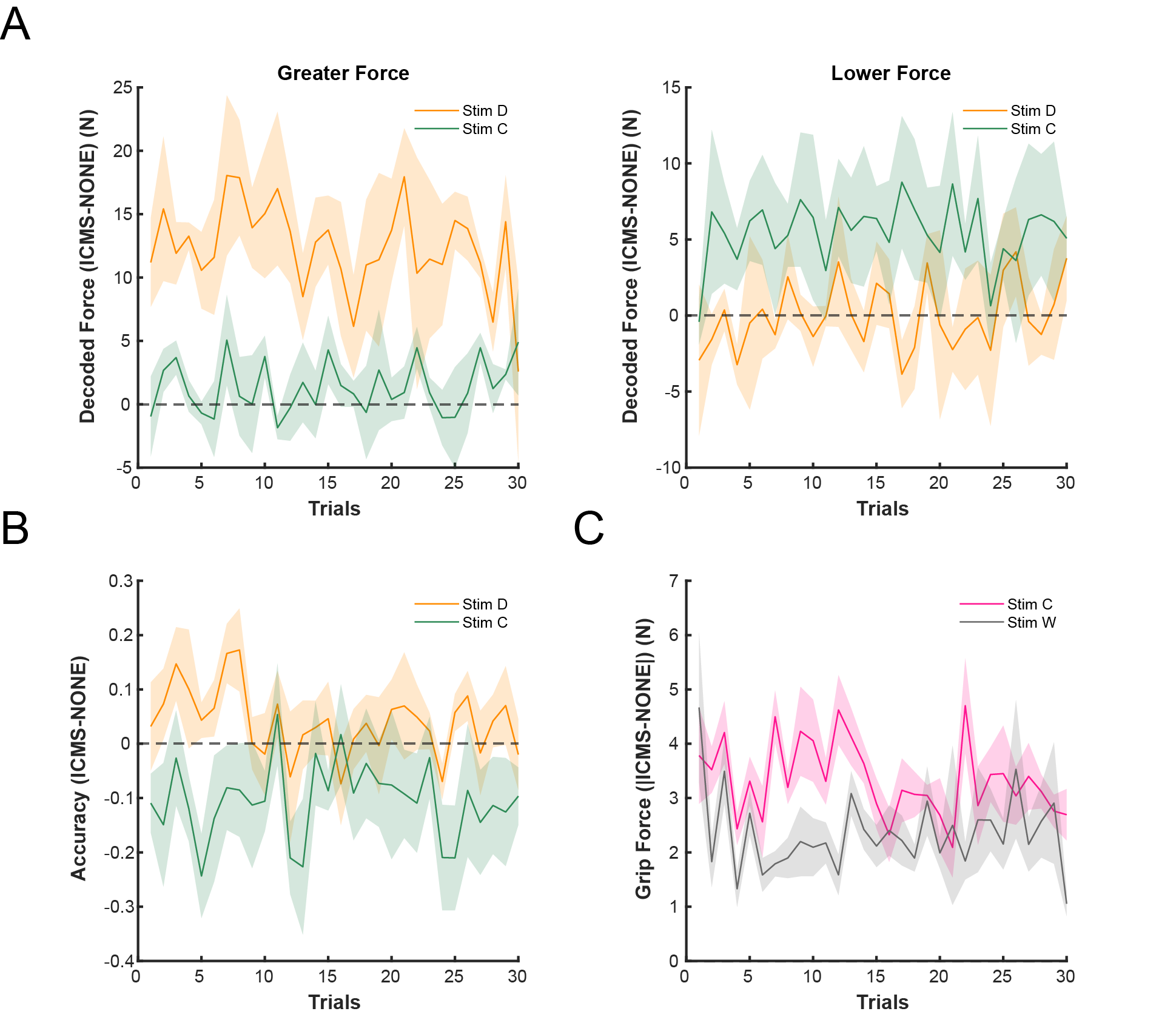
**

**Figure S7. Within-Block Neural Adaptation Effects of ICMS on Neural Decoding and Grip Force, Related to Figures 6–8. (A)** Grip force decoding bias under ICMS throughout all within-block 30 trials. Stimulus D consistently increases decoded force under greater-force tactile conditions (left), while stimulus C increases decoded force under lower-force conditions (right). No evidence of neural adaptation is observed. **(B)** Classification accuracy bias under ICMS throughout all within-block 30 trials. Stimulus D reliably improves accuracy, whereas stimulus C reduces accuracy. Both effects remain stable over time. **(C)** Actual grip force bias under ICMS throughout all within-block 30 trials. Stimulus C induces a consistent behavioral shift with no sign of adaptation.


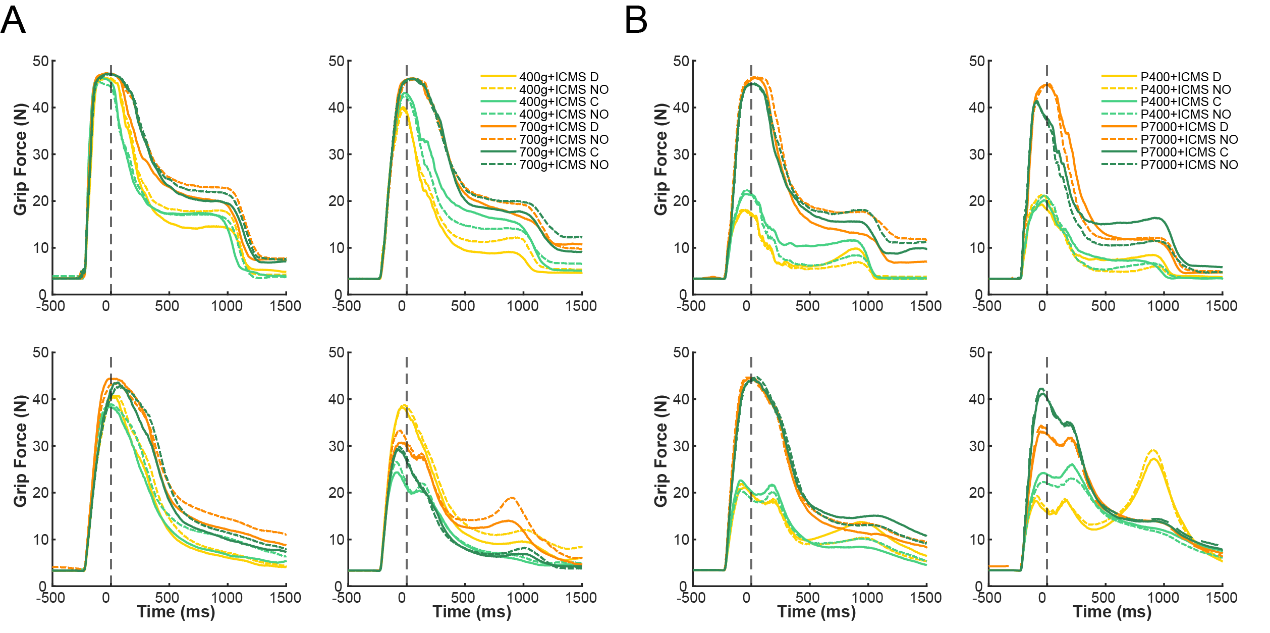


**Figure S8. Modulation of Stimuli C and D on Actual Grip Forces, Related to Figure 8.** (**A**) Trial-averaged grip forces for all sessions of varied load levels. Effects of ICMS on force appear from 300 ms to 1000 ms after the onset of stimulation. Solid lines correspond to forces in trials with stimulation, while dashed lines represent force trials without stimulation. The upper panels display data from Monkey A, and the lower panels show data from Monkey B. (**B**) Trial-averaged grip forces for all sessions of various texture levels.


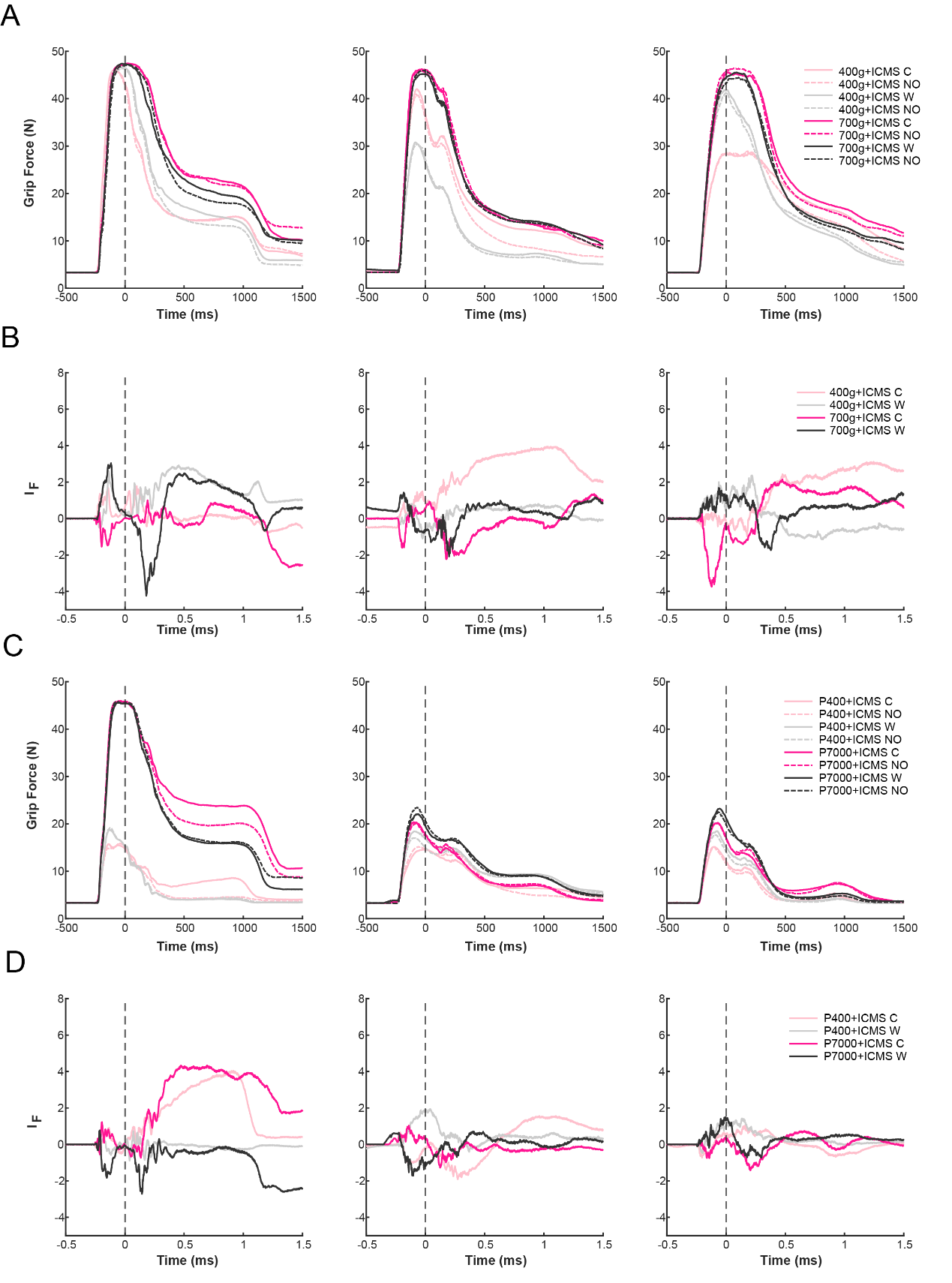


**Figure S9. Modulation of Stimuli C and W on Actual Grip Force, Related to Figure 8.** (**A**) Trial-averaged grip forces for the rest sessions of varied load levels. The panel on the left displays data from Monkey A, and the right two panels display data from Monkey B. (**B**) Trial-averaged index of difference for grip force (I_F_) for the rest sessions of varied load levels. Stimulus C shows a greater effect on force modulation, suggesting ICMS encoded by S1→M1 interaction is effective in behavioral modulation in grasping. (**C**) Alike (**A**), trial-averaged grip forces for sessions of varied texture levels. (**D**) Alike (**B**), trial-averaged I_F_ for sessions of various texture levels.
